# Supplementary figures and images for: The network structure of hematopoietic cancers
Source: Sci Rep. 2023 Nov 13;13:19837. doi: 10.1038/s41598-023-46655-2 (PMC10645882; doi:10.1038/s41598-023-46655-2)

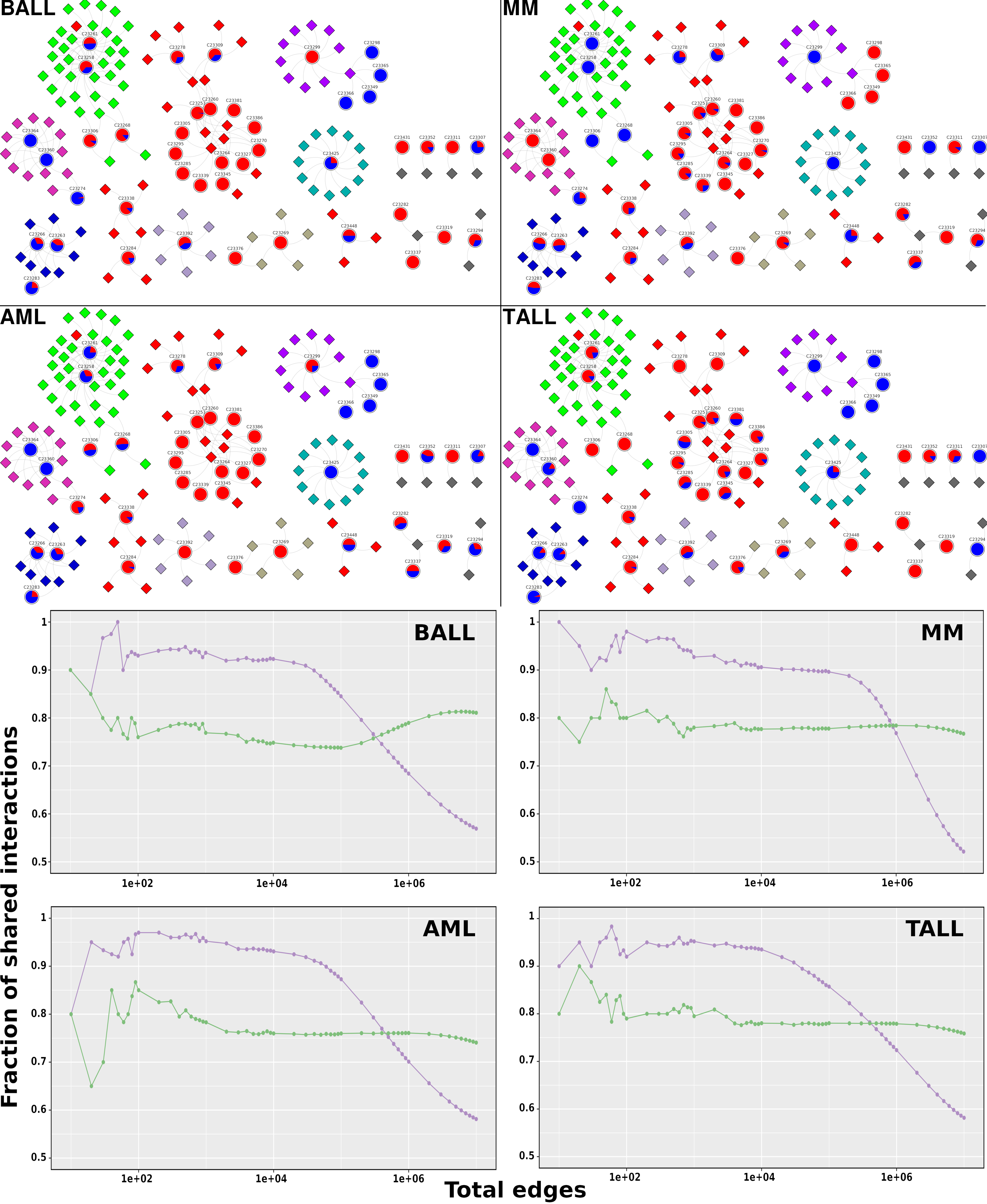

Supplement: Supplementary file 4 — Supplementary Information 4. [file 41598_2023_46655_MOESM4_ESM.png]
