# Supplementary material for: The network structure of hematopoietic cancers
Source: Sci Rep. 2023 Nov 13;13:19837. doi: 10.1038/s41598-023-46655-2 (PMC10645882; doi:10.1038/s41598-023-46655-2)

Quality Control of Expression Data
AML Before Normalization

Generated by NOISeq on 21 Nov 2022, 22:01:41

Content

Plot	*Description*
**Biotype detection**	Biotype abundance in the genome with %genes detected (counts > 0) in the sample/condition.<br>Biotype abundance within the sample/condition.
**Biotype expression**	Distribution of gene counts per million per biotype in sample/condition (only genes with counts > 0).
**Saturation**	Number of detected genes (counts > 0) per sample across different sequencing depths
**Expression boxplot**	Distribution of gene counts per million (all biotypes) in each sample/condition
**Expression barplot**	Percentage of genes with >0, >1, >2, >5 or >10 counts per million in each sample/condition.
**Length bias**	Mean gene expression per each length bin. Fitted curve and diagnostic test.
**GC content bias**	Mean gene expression per each GC content bin. Fitted curve and diagnostic test.
**RNA composition bias**	Density plots of log fold changes (M) between pairs of samples.<br>Confidence intervals for the median of M values.
**Exploratory PCA**	Principal Component Analysis score plots for PC1 vs PC2, and PC1 vs PC3.

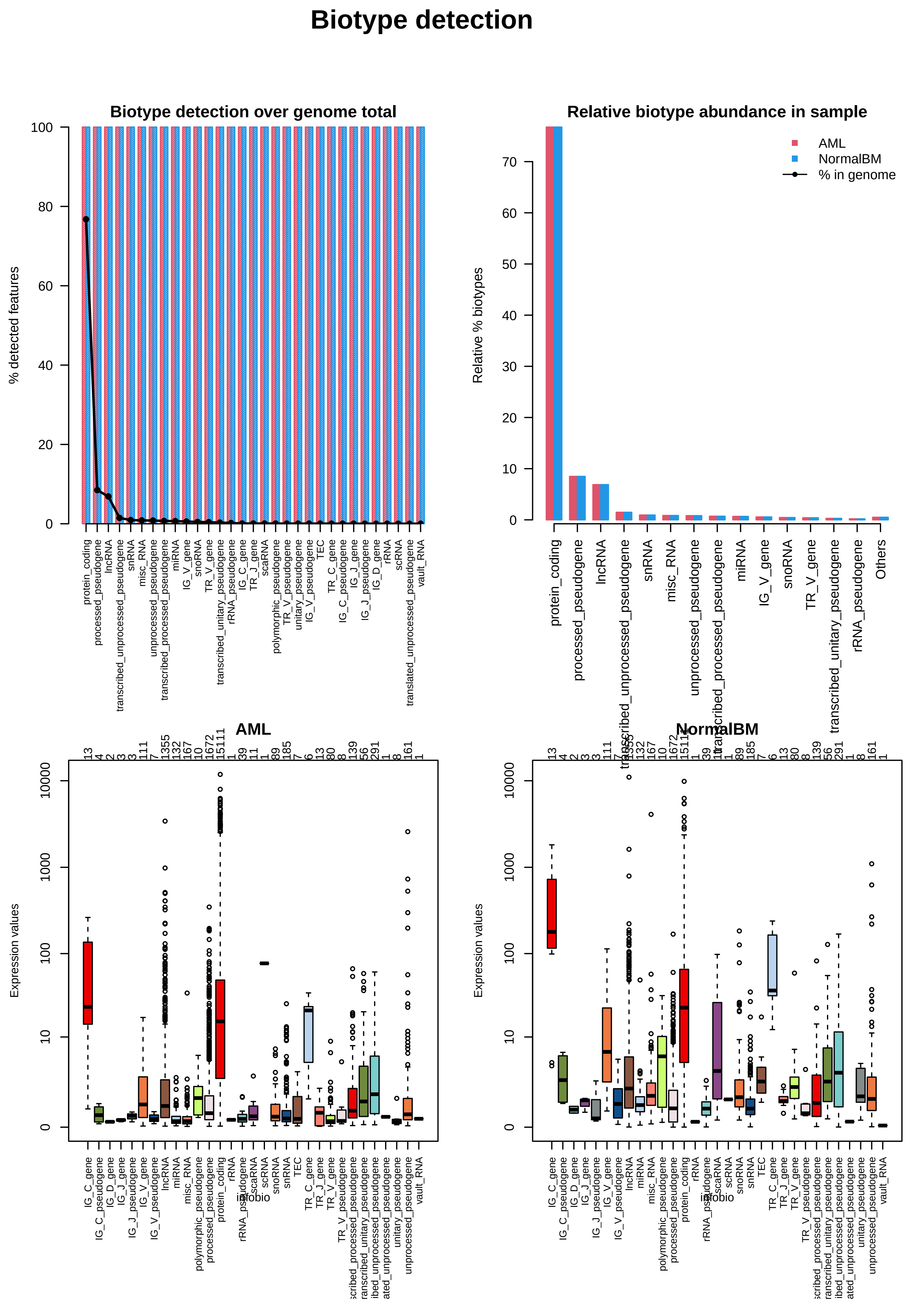
Biotype detection
Biotype detection over genome total
% detected features
protein_coding
processed_pseudogene
lncRNA
transcribed_unprocessed_pseudogene
snRNA
misc_RNA
unprocessed_pseudogene
transcribed_processed_pseudogene
miRNA
IG_V_gene
snoRNA
TR_V_gene
transcribed_unitary_pseudogene
rRNA_pseudogene
IG_C_gene
TR_J_gene
scaRNA
polymorphic_pseudogene
TR_V_pseudogene
unitary_pseudogene
IG_V_pseudogene
TEC
TR_C_gene
IG_C_pseudogene
IG_J_gene
IG_J_pseudogene
IG_D_gene
rRNA
scRNA
translated_unprocessed_pseudogene
vault_RNA
Relative biotype abundance in sample
Relative % biotypes
AML
NormalBM
% in genome
Others
AML
NormalBM
Expression values
infobio

Sequencing depth & Expression quantification

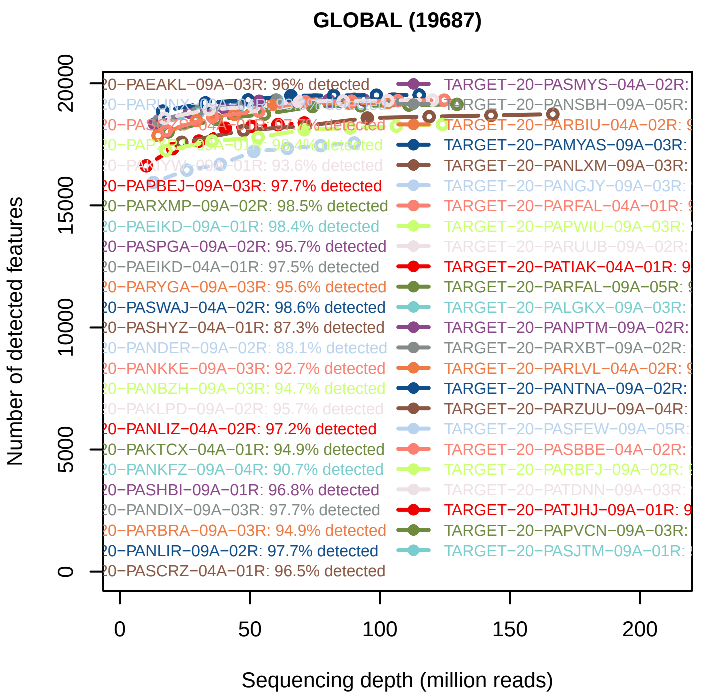

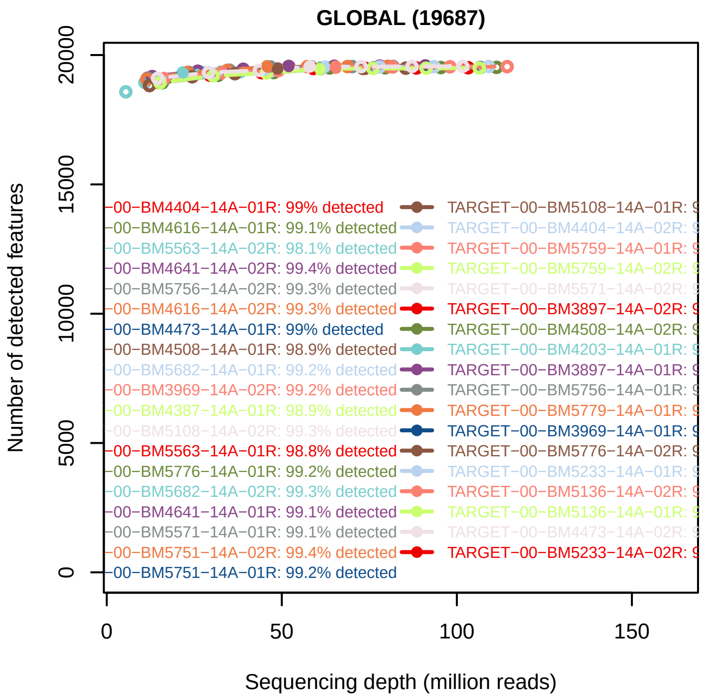

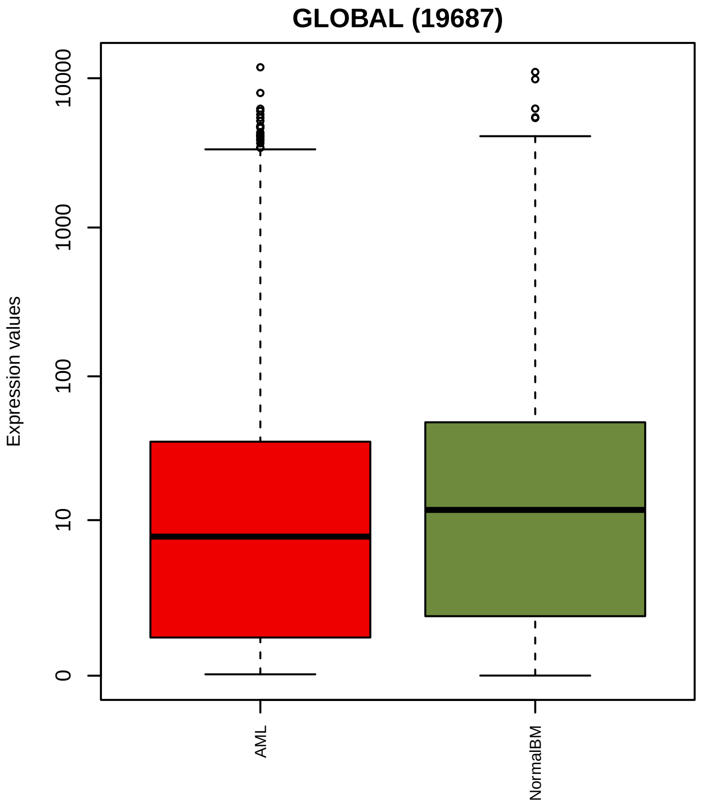

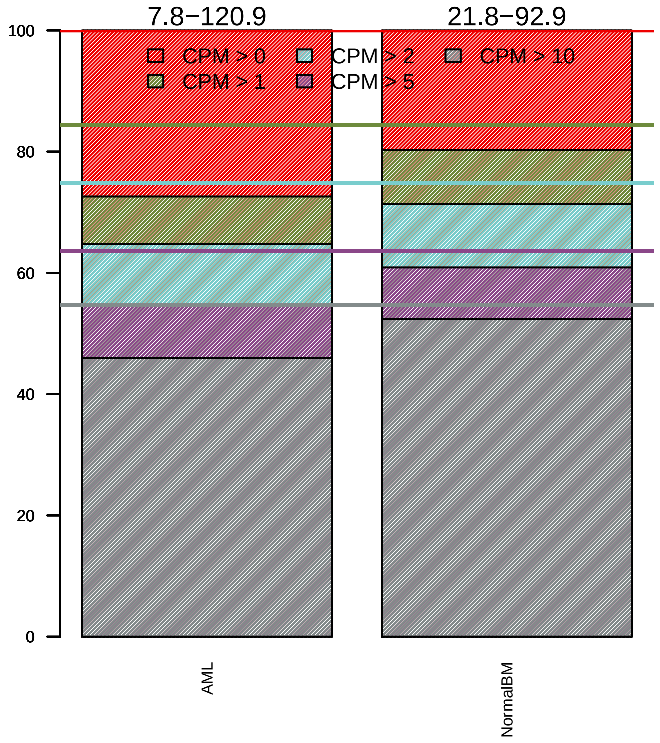

Sequencing bias detection

Diagnostic plot for feature length bias

FAILED. At least one of the model p−values was lower than 0.05 and R2 > 70%.

Normalization for correcting length bias is recommended.

AML

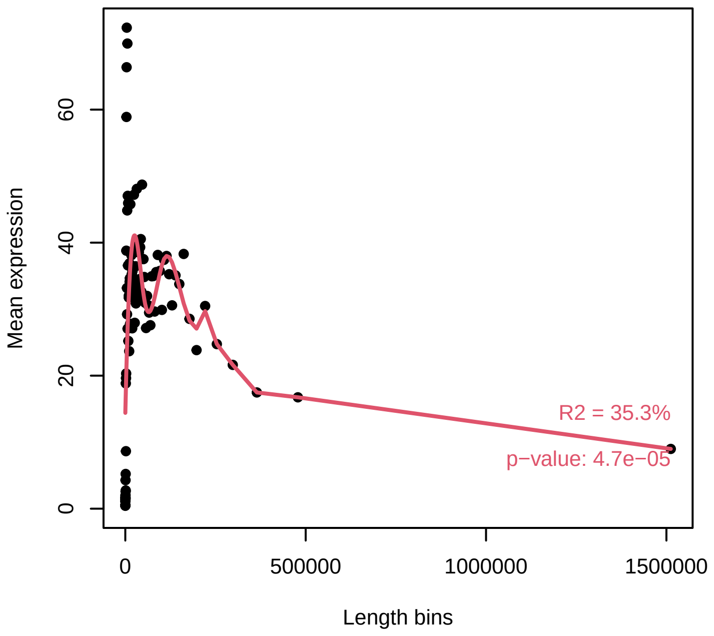

NormalBM

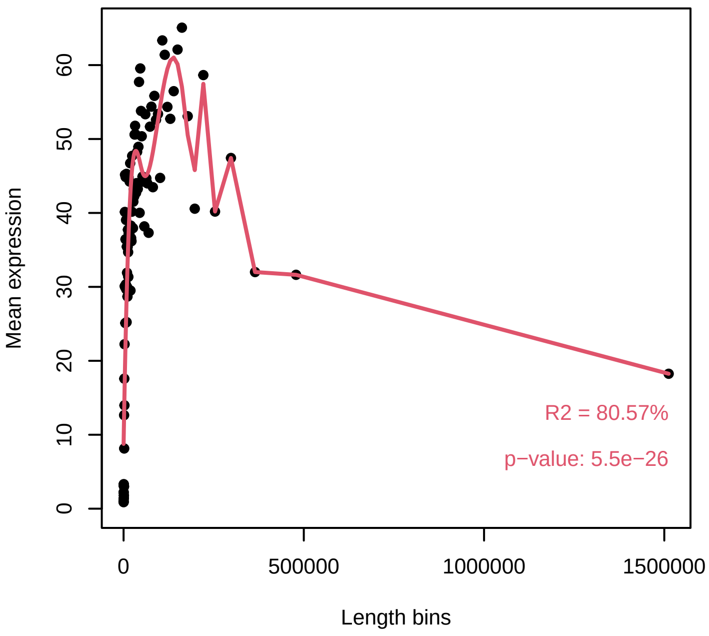

Diagnostic plot for GC content bias

WARNING. At least one of the model p−values was lower than 0.05, but R2 < 70% for at least one condition.

Normalization for correcting GC content bias could be advisable.
Plese check in the plots below the strength of the relationship between GC content and expression.

AML

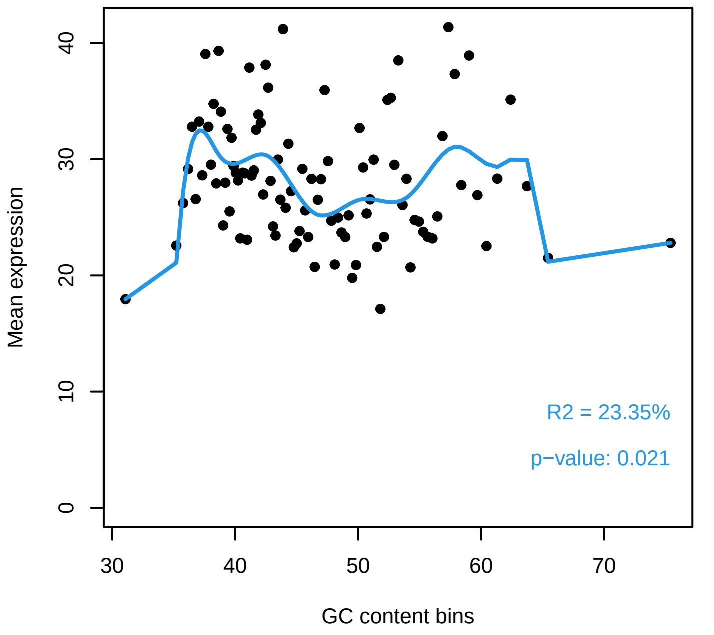

NormalBM

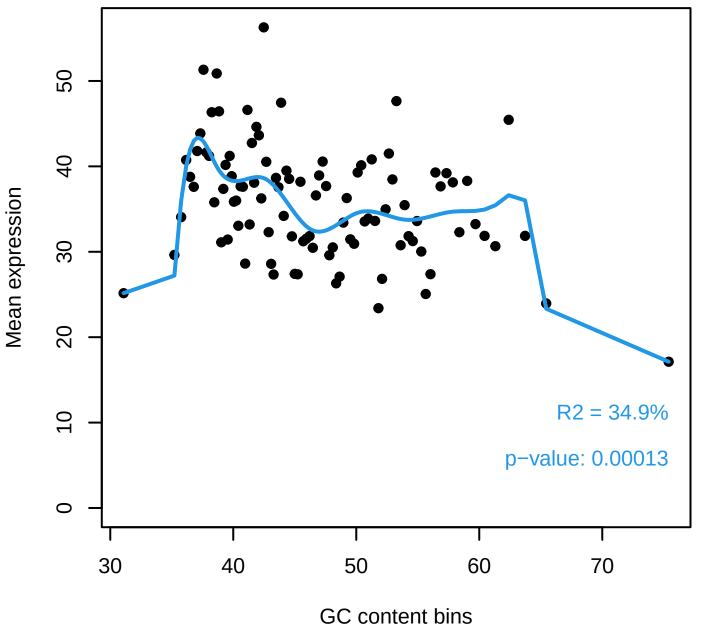

Diagnostic plot for differences in RNA composition

FAILED. There is a pair of samples with significantly different RNA composition

Normalization for correcting this bias is required.

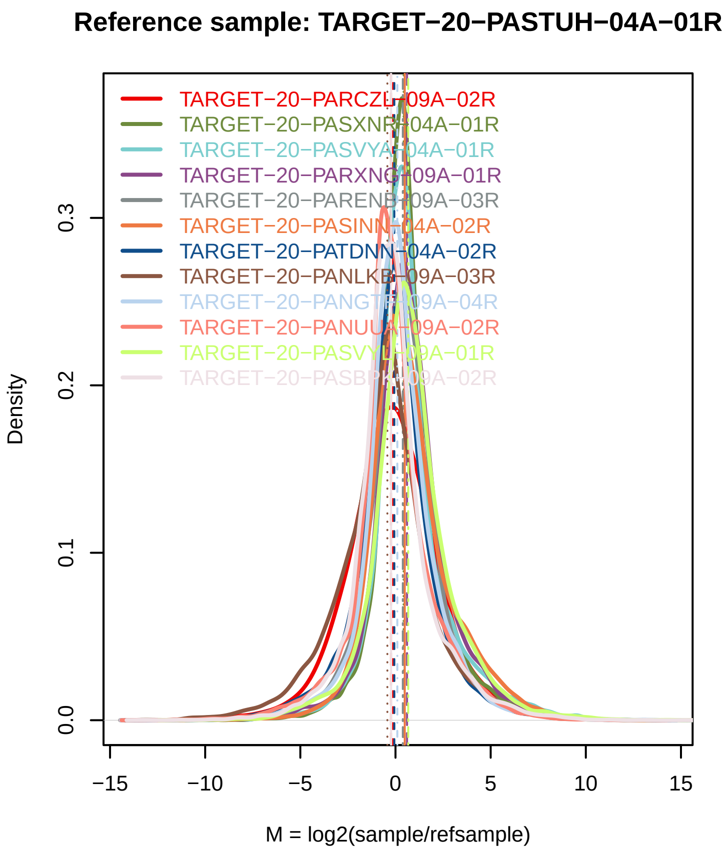

Confidence intervals for median of M values

Sample	0.01%	99.99%	Diagnostic Test
TARGET−20−PARCZL−09A−02R	−0.1953	−0.0681	FAILED
TARGET−20−PASXNR−04A−01R	0.4254	0.4462	FAILED
TARGET−20−PASVYA−04A−01R	0.4435	0.5103	FAILED
TARGET−20−PARXNC−09A−01R	0.5599	0.6396	FAILED
TARGET−20−PARENB−09A−03R	0.3379	0.434	FAILED
TARGET−20−PASINN−04A−02R	0.4388	0.5309	FAILED
TARGET−20−PATDNN−04A−02R	−0.1142	−0.0113	FAILED
TARGET−20−PANLKB−09A−03R	−0.4841	−0.3519	FAILED
TARGET−20−PANGTF−09A−04R	0.0605	0.1387	FAILED
TARGET−20−PANUUA−09A−02R	−0.2765	−0.204	FAILED
TARGET−20−PASVYL−09A−01R	0.0631	0.7148	FAILED
TARGET−20−PASBPK−09A−02R	−0.2784	−0.1903	FAILED
TARGET−20−PARAJX−09A−02R	−0.5092	−0.385	FAILED
TARGET−20−PANVGE−09A−02R	−0.8399	−0.7944	FAILED
TARGET−20−PASTTW−09A−01R	0.5479	0.6531	FAILED
TARGET−20−PARCUK−09A−03R	−0.2715	−0.2608	FAILED
TARGET−20−PASIBG−09A−01R	0.4532	0.531	FAILED
TARGET−20−PARUNX−09A−01R	0.445	0.5293	FAILED
TARGET−20−PASVVS−09A−03R	0.0113	0.0812	FAILED
TARGET−20−PASXYG−09A−05R	−0.0397	0.0555	PASSED
TARGET−20−PARBIU−09A−03R	0.5912	0.5977	FAILED
TARGET−20−PAPXWI−09A−03R	−0.7941	−0.7891	FAILED
TARGET−20−PARTAL−09A−01R	0.3967	0.4631	FAILED
TARGET−20−PARHVK−09A−04R	0.16	0.2469	FAILED
TARGET−20−PASCFW−09A−01R	0.0609	0.157	FAILED
TARGET−20−PAPWYK−10A−03R	−0.1097	−0.0521	FAILED
TARGET−20−PARCZL−04A−02R	0.2297	0.2999	FAILED
TARGET−20−PARUTH−09A−04R	−0.3924	−0.2891	FAILED

Exploratory PCA

Use this plot to see if samples are clustered according to the experimental design.

Use ARSyNseq function to correct potential batch effects.

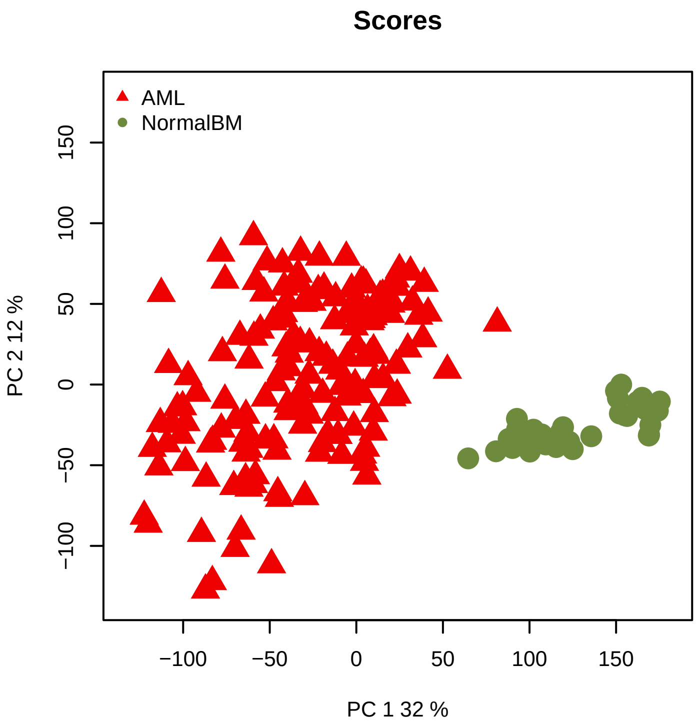

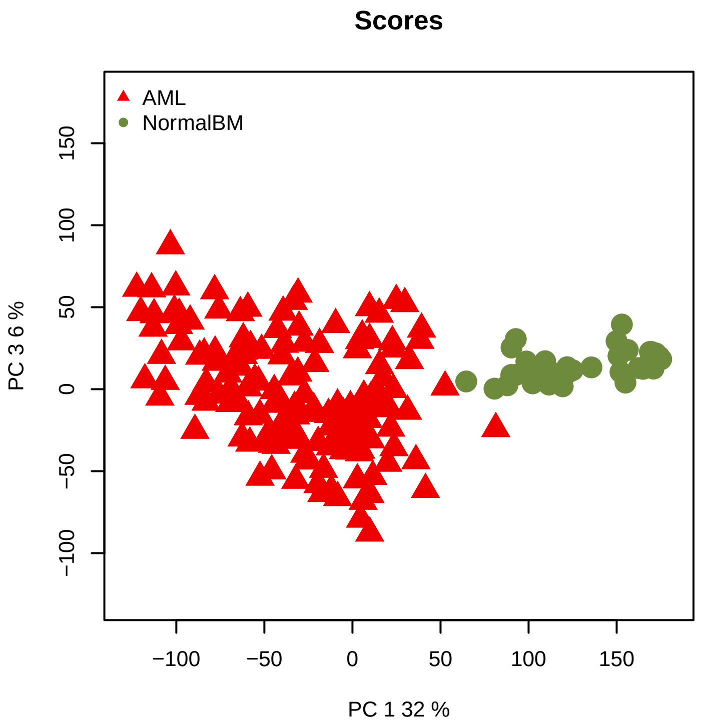

Quality Control of Expression Data
AML After Normalization

Generated by NOISeq on 22 Nov 2022, 00:15:02

Content

Plot	*Description*
**Biotype detection**	Biotype abundance in the genome with %genes detected (counts > 0) in the sample/condition.<br>Biotype abundance within the sample/condition.
**Biotype expression**	Distribution of gene counts per million per biotype in sample/condition (only genes with counts > 0).
**Saturation**	Number of detected genes (counts > 0) per sample across different sequencing depths
**Expression boxplot**	Distribution of gene counts per million (all biotypes) in each sample/condition
**Expression barplot**	Percentage of genes with >0, >1, >2, >5 or >10 counts per million in each sample/condition.
**Length bias**	Mean gene expression per each length bin. Fitted curve and diagnostic test.
**GC content bias**	Mean gene expression per each GC content bin. Fitted curve and diagnostic test.
**RNA composition bias**	Density plots of log fold changes (M) between pairs of samples.<br>Confidence intervals for the median of M values.
**Exploratory PCA**	Principal Component Analysis score plots for PC1 vs PC2, and PC1 vs PC3.

Biotype detection

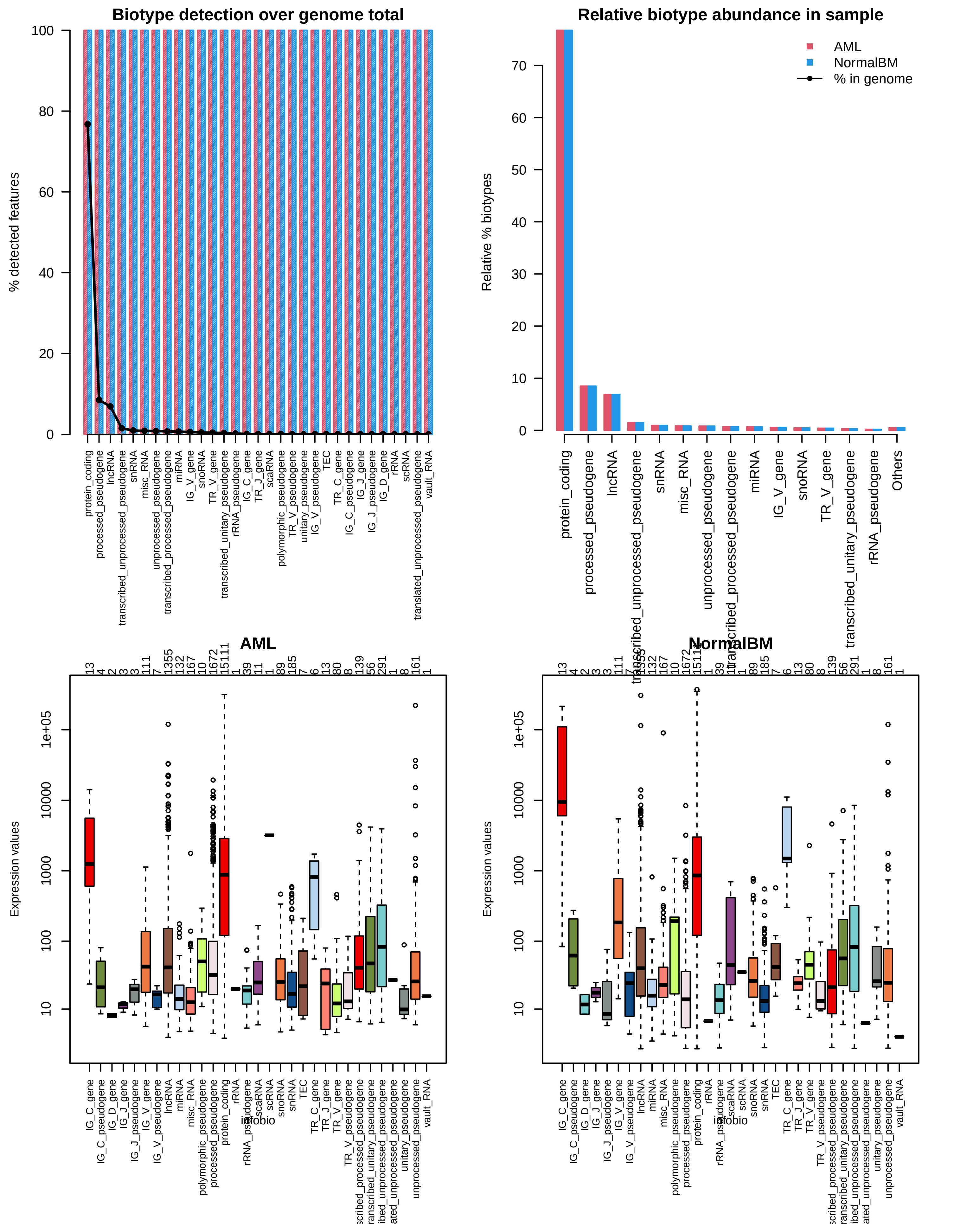

Sequencing depth & Expression quantification

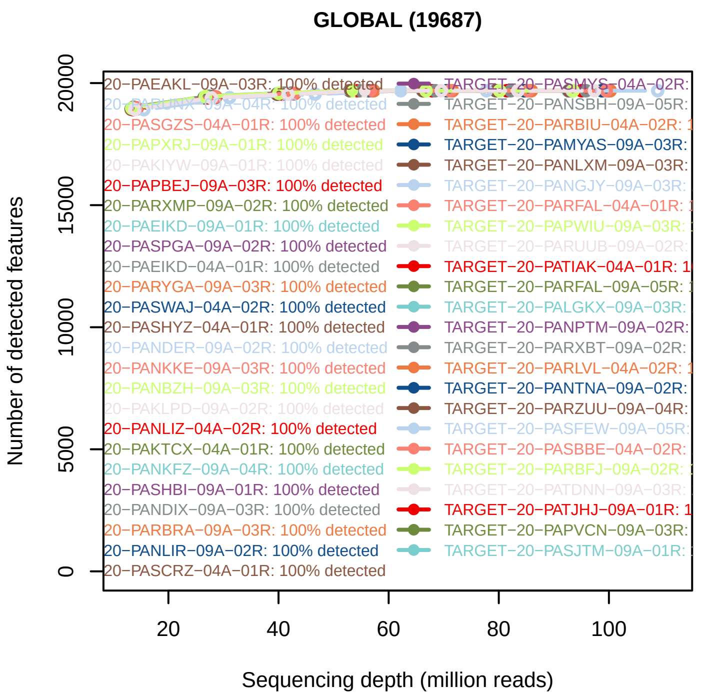

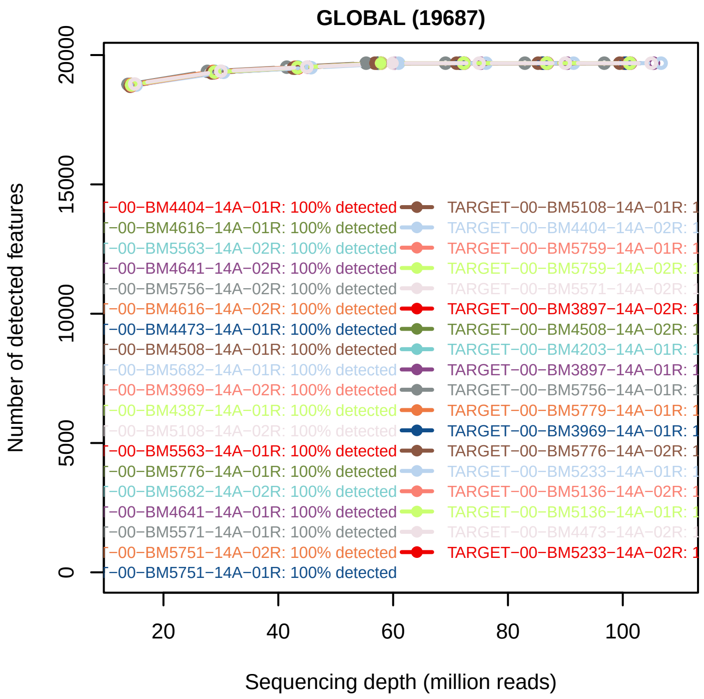

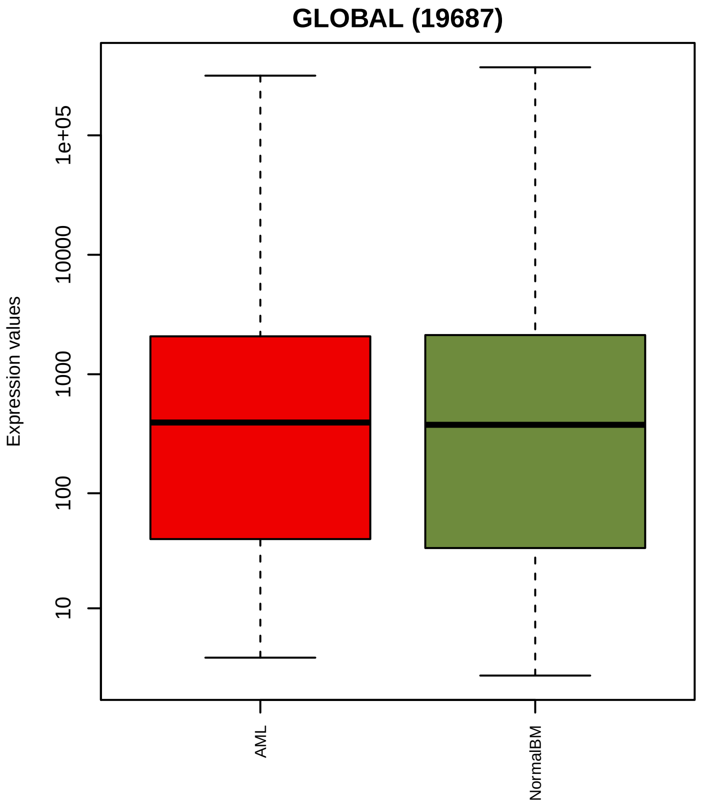

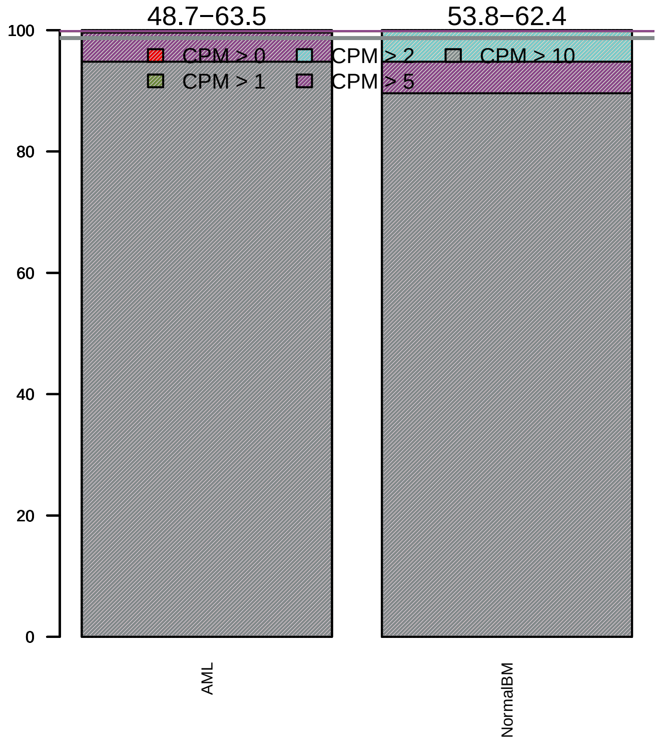

Sequencing bias detection

Diagnostic plot for feature length bias

FAILED. At least one of the model p−values was lower than 0.05 and R2 > 70%.

Normalization for correcting length bias is recommended.

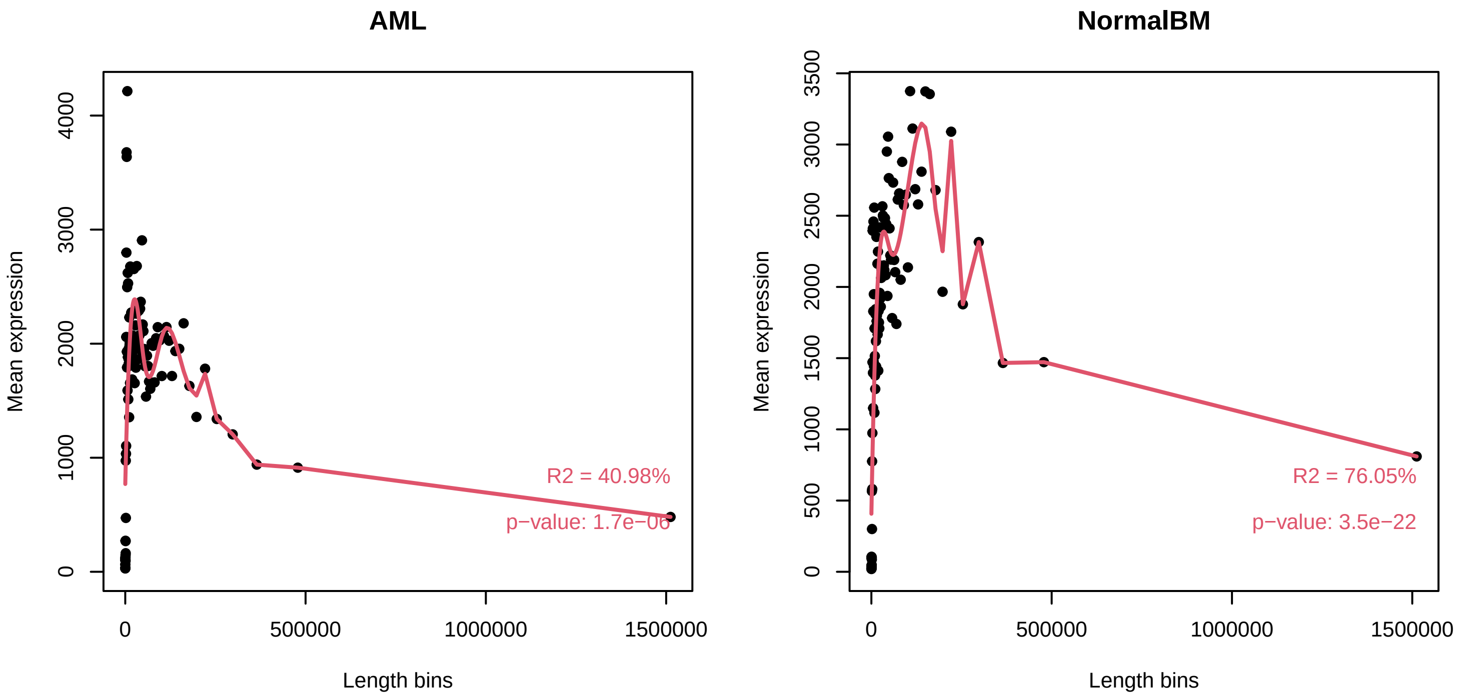

Diagnostic plot for GC content bias

WARNING. At least one of the model p−values was lower than 0.05, but R2 < 70% for at least one condition.

Normalization for correcting GC content bias could be advisable.
Plese check in the plots below the strength of the relationship between GC content and expression.

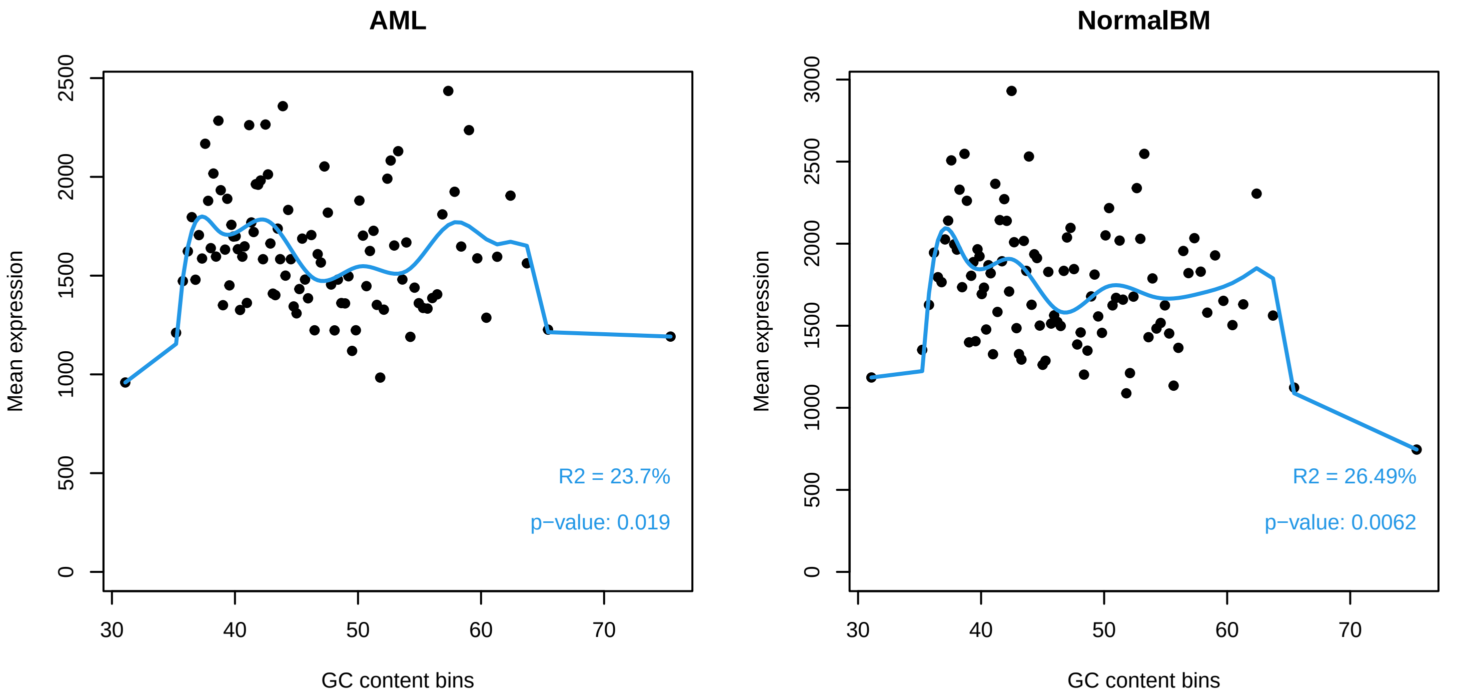

Diagnostic plot for differences in RNA composition

FAILED. There is a pair of samples with significantly different RNA composition

Normalization for correcting this bias is required.

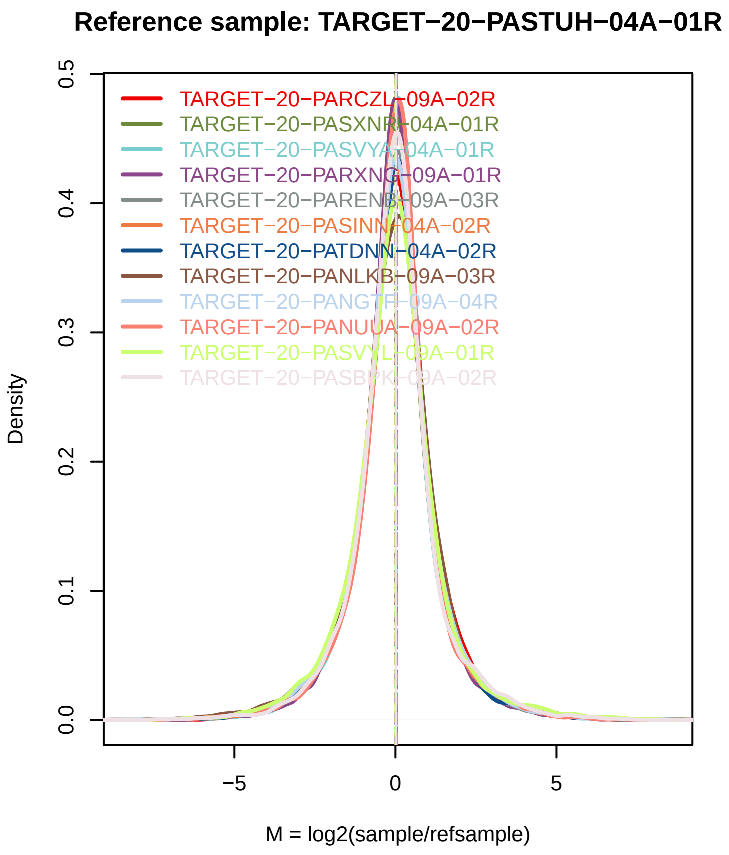

Confidence intervals for median of M values

Sample	0.01%	99.99%	Diagnostic Test
TARGET−20−PARCZL−09A−02R	−0.0098	0.0487	PASSED
TARGET−20−PASXNR−04A−01R	−0.0271	0.0212	PASSED
TARGET−20−PASVYA−04A−01R	−0.005	0.0425	PASSED
TARGET−20−PARXNG−09A−01R	−0.0175	0.0248	PASSED
TARGET−20−PARENB−09A−03R	0.0192	0.0665	FAILED
TARGET−20−PASINN−04A−02R	−0.0062	0.0383	PASSED
TARGET−20−PATDNN−04A−02R	0.0021	0.0487	FAILED
TARGET−20−PANLKB−09A−03R	0.0101	0.0638	FAILED
TARGET−20−PANGTF−09A−04R	−0.0053	0.041	PASSED
TARGET−20−PANUUA−09A−02R	0.0186	0.0591	FAILED
TARGET−20−PASVYL−09A−01R	−0.0239	0.0325	PASSED
TARGET−20−PASBPK−09A−02R	−0.0217	0.032	PASSED
TARGET−20−PARAJX−09A−02R	0.0421	0.0876	FAILED
TARGET−20−PANVGE−09A−02R	−0.086	−0.0286	FAILED
TARGET−20−PASTTW−09A−01R	−0.0108	0.0395	PASSED
TARGET−20−PARCUK−09A−03R	−0.0022	0.0494	PASSED
TARGET−20−PASIBG−09A−01R	−0.0113	0.04	PASSED
TARGET−20−PARUNX−09A−01R	0.0052	0.0476	FAILED
TARGET−20−PASVVS−09A−03R	0.0153	0.0648	FAILED
TARGET−20−PASXYG−09A−05R	0.0031	0.06	FAILED
TARGET−20−PARBIU−09A−03R	−0.0019	0.0433	PASSED
TARGET−20−PAPXWI−09A−03R	−0.0717	−0.0183	FAILED
TARGET−20−PARTAL−04A−01R	0.0205	0.0548	FAILED
TARGET−20−PARHVK−09A−04R	0.0174	0.0703	FAILED
TARGET−20−PASCFW−09A−01R	0.0391	0.0908	FAILED
TARGET−20−PAPWYK−09A−03R	0.0107	0.0603	FAILED
TARGET−20−PARCZL−09A−02R	−0.0095	0.0428	PASSED
TARGET−20−PARUTH−09A−04R	0.0138	0.0656	FAILED

Exploratory PCA

Use this plot to see if samples are clustered according to the experimental design.

Use ARSyNseq function to correct potential batch effects.

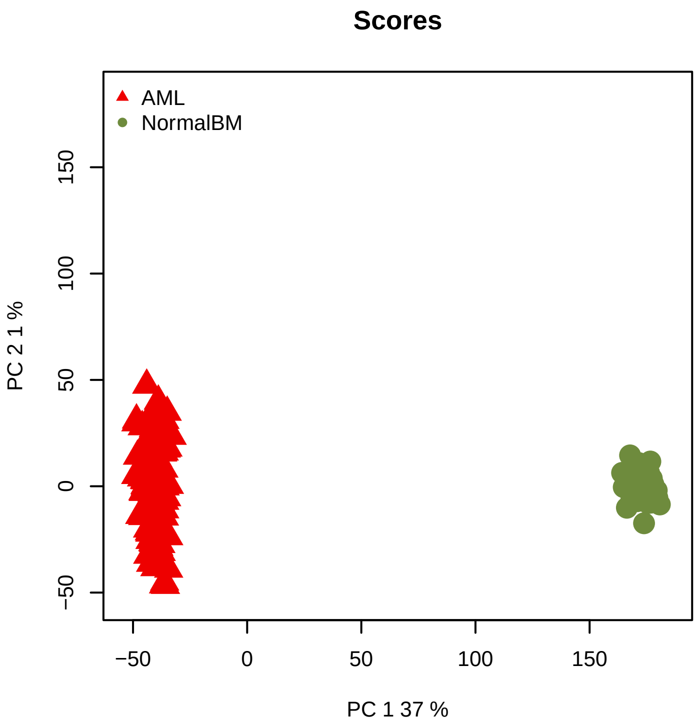

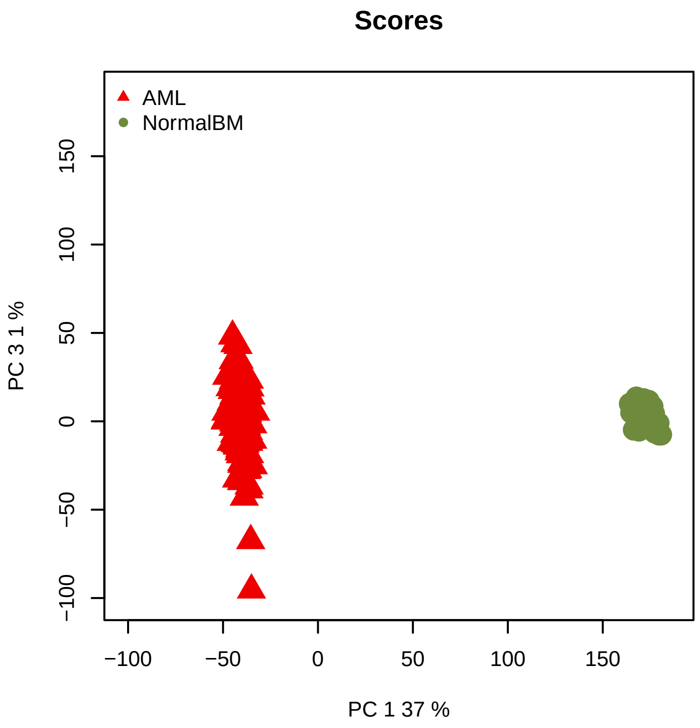

Quality Control of Expression Data
BALL Before Normalization

Generated by NOISeq on 21 Nov 2022, 22:29:20

Content

Plot	*Description*
**Biotype detection**	Biotype abundance in the genome with %genes detected (counts > 0) in the sample/condition.<br>Biotype abundance within the sample/condition.
**Biotype expression**	Distribution of gene counts per million per biotype in sample/condition (only genes with counts > 0).
**Saturation**	Number of detected genes (counts > 0) per sample across different sequencing depths
**Expression boxplot**	Distribution of gene counts per million (all biotypes) in each sample/condition
**Expression barplot**	Percentage of genes with >0, >1, >2, >5 or >10 counts per million in each sample/condition.
**Length bias**	Mean gene expression per each length bin. Fitted curve and diagnostic test.
**GC content bias**	Mean gene expression per each GC content bin. Fitted curve and diagnostic test.
**RNA composition bias**	Density plots of log fold changes (M) between pairs of samples.<br>Confidence intervals for the median of M values.
**Exploratory PCA**	Principal Component Analysis score plots for PC1 vs PC2, and PC1 vs PC3.

Biotype detection

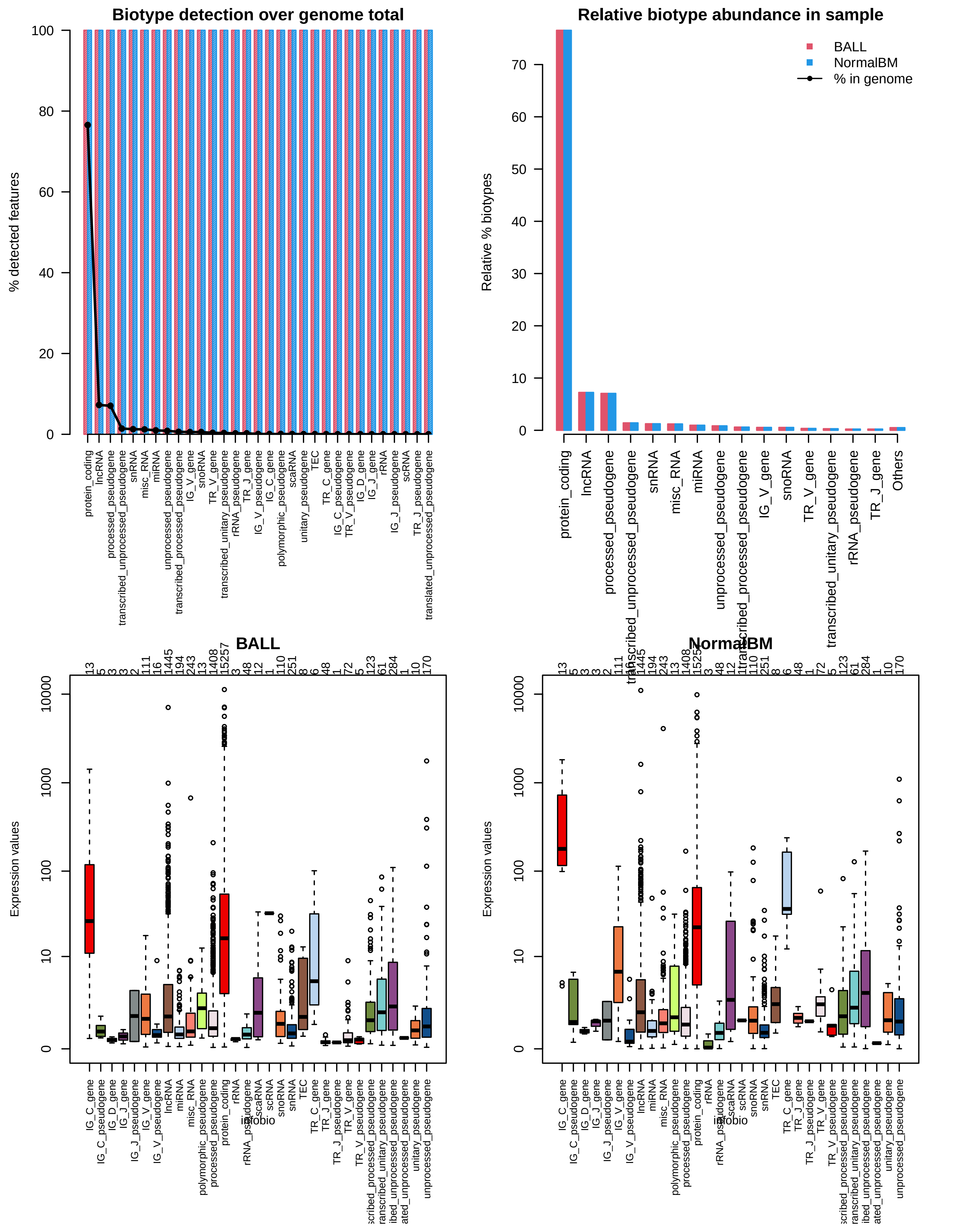

Sequencing depth & Expression quantification

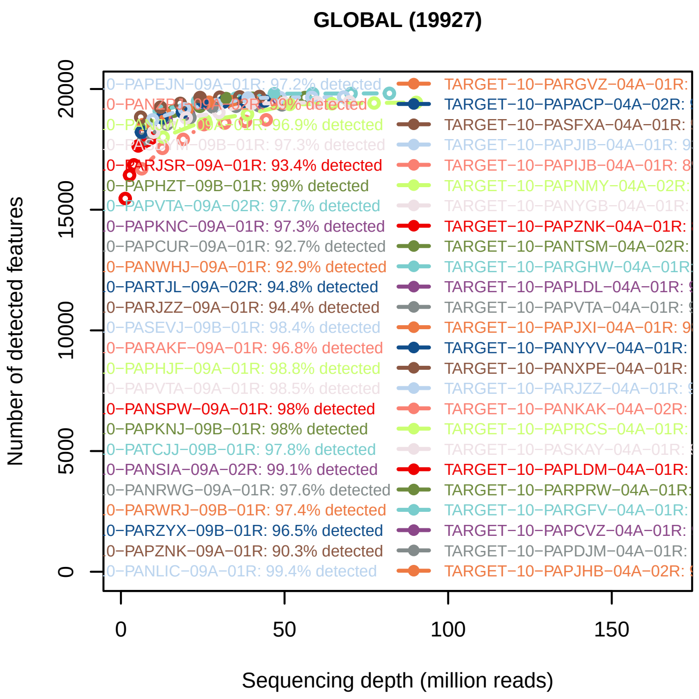

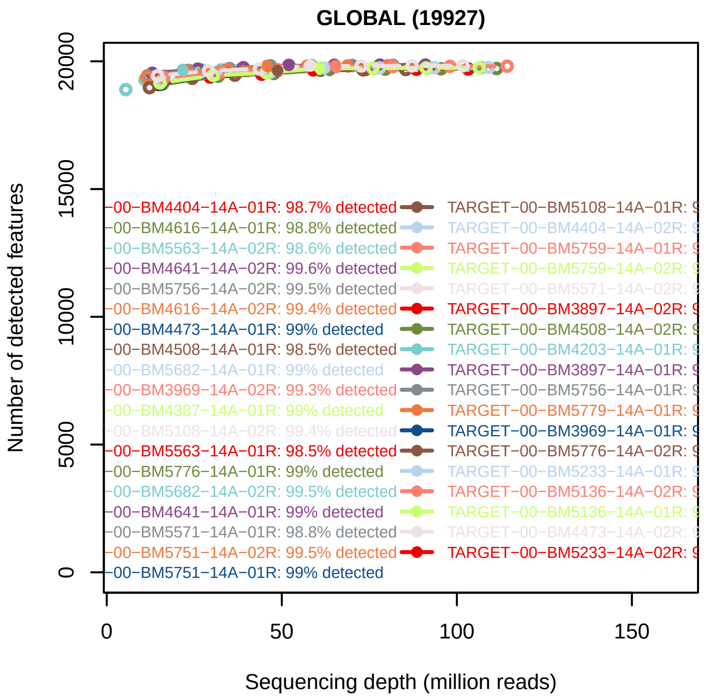

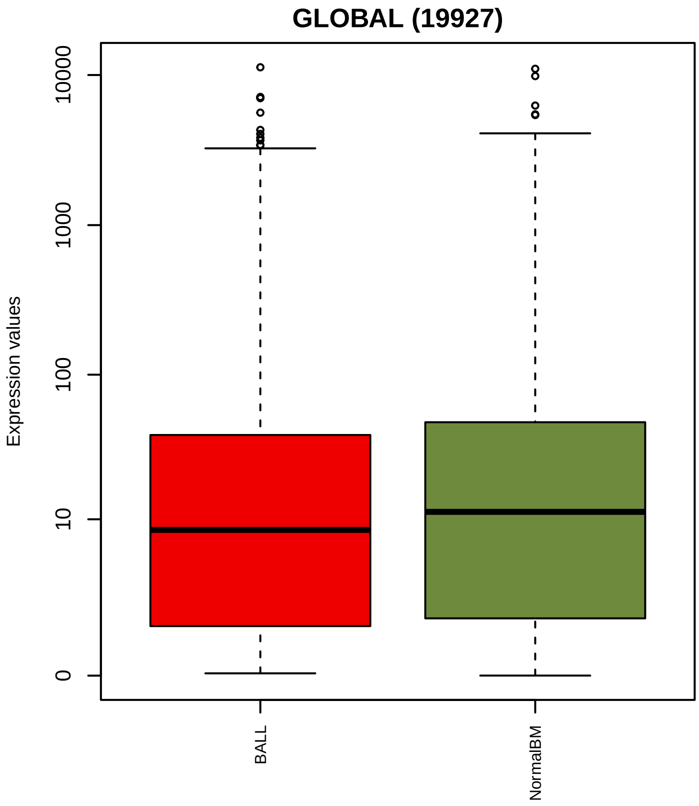

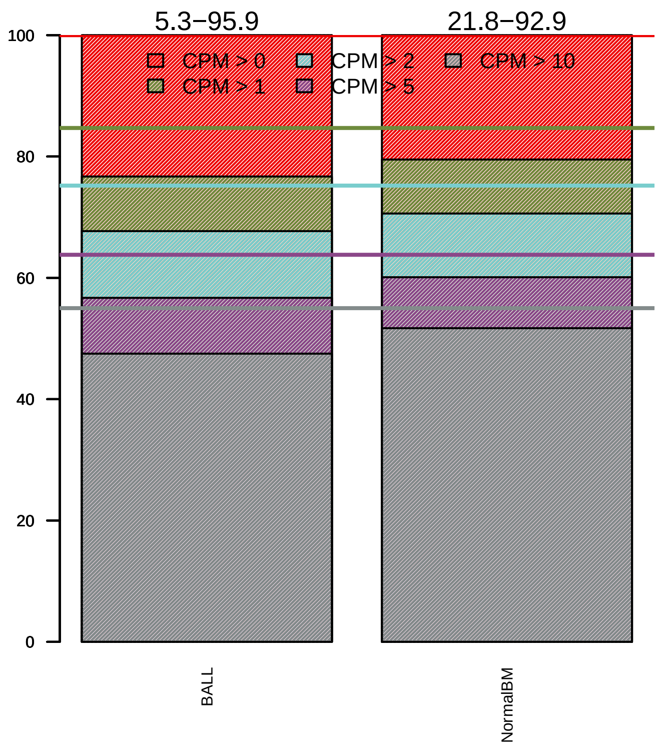

Sequencing bias detection

Diagnostic plot for feature length bias

FAILED. At least one of the model p−values was lower than 0.05 and R2 > 70%.

Normalization for correcting length bias is recommended.

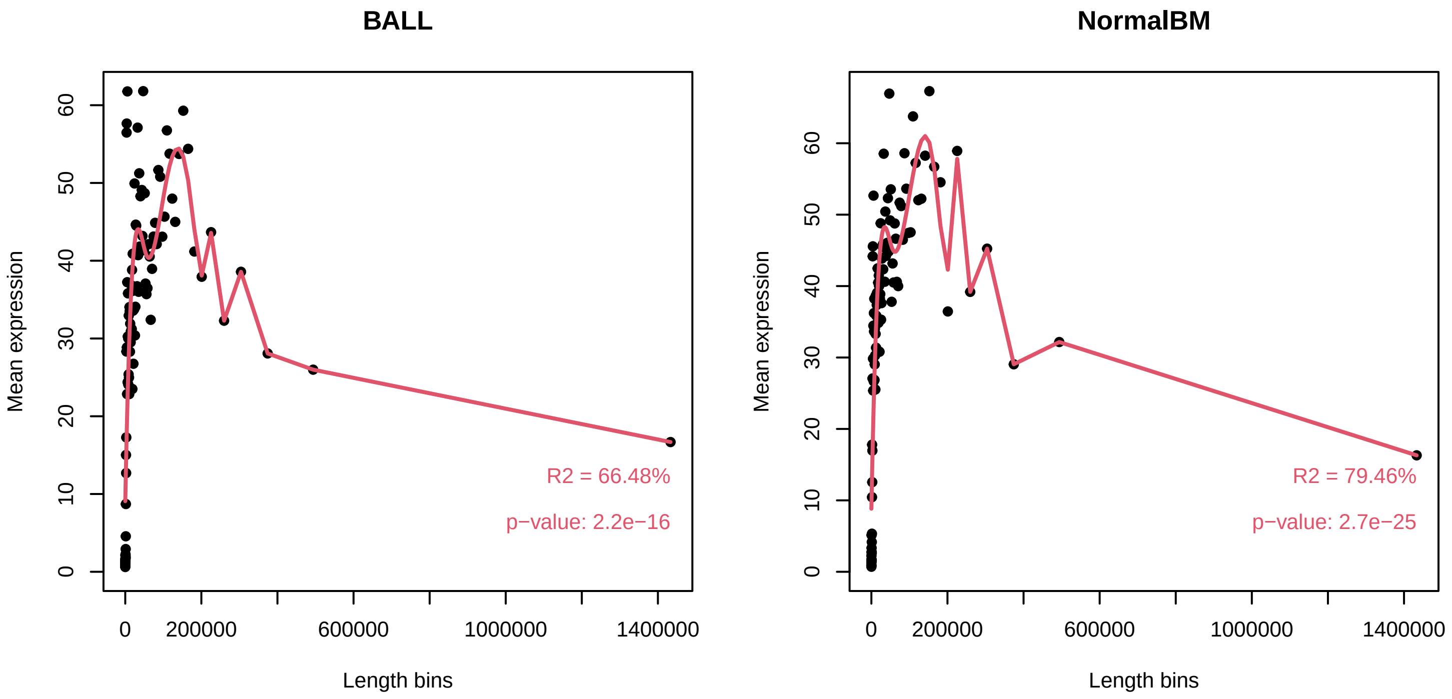

Diagnostic plot for GC content bias

WARNING. At least one of the model p−values was lower than 0.05, but R2 < 70% for at least one condition.

Normalization for correcting GC content bias could be advisable.
Plese check in the plots below the strength of the relationship between GC content and expression.

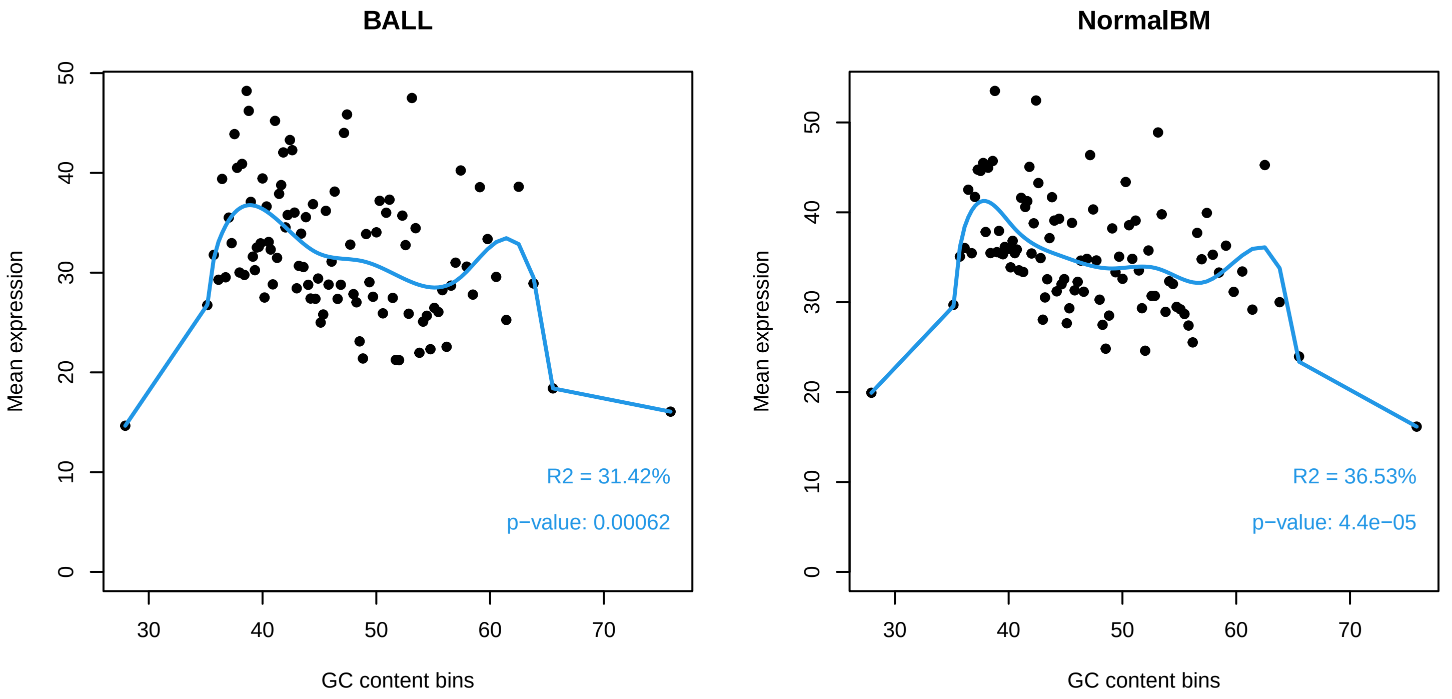

Diagnostic plot for differences in RNA composition

FAILED. There is a pair of samples with significantly different RNA composition

Normalization for correcting this bias is required.

Reference sample: TARGET−10−PARBVI−09A−02R

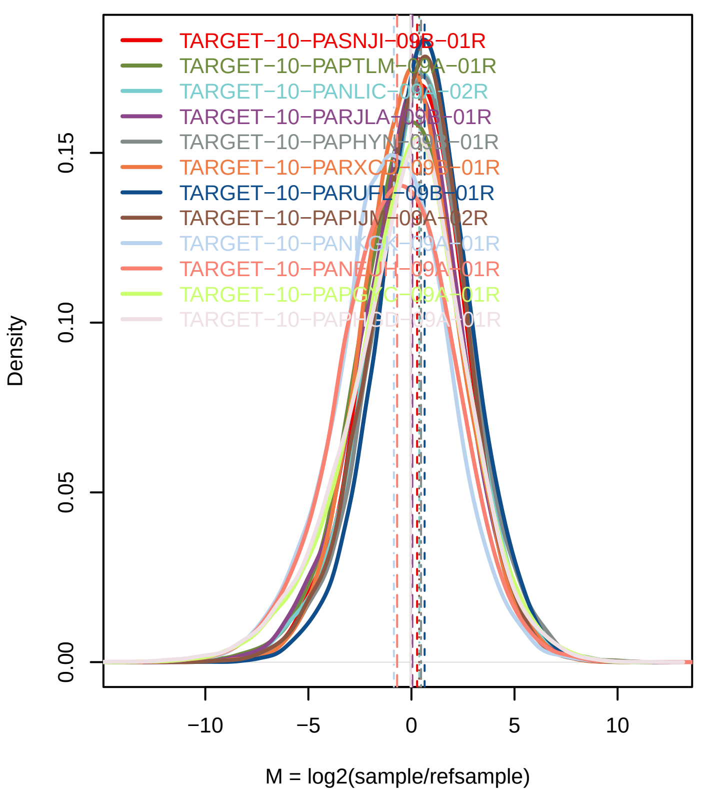

Confidence intervals for median of M values

Sample	0.01%	99.99%	Diagnostic Test
TARGET−10−PASNJI−09B−01R	0.1987	0.3451	FAILED
TARGET−10−PAPTLM−09A−01R	−0.1087	0.0468	PASSED
TARGET−10−PANLIC−09A−02R	0.2959	0.4375	FAILED
TARGET−10−PARJLA−03B−01R	−0.0359	0.12	PASSED
TARGET−10−PAPHYN−09B−01R	0.4016	0.5462	FAILED
TARGET−10−PARXCD−09B−01R	−0.0113	0.0651	PASSED
TARGET−10−PARUFL−09B−01R	0.5701	0.7009	FAILED
TARGET−10−PAPIJM−09A−02R	0.2872	0.4287	FAILED
TARGET−10−PANKGK−09A−01R	−0.9048	−0.7626	FAILED
TARGET−10−PANEUH−09A−01R	−0.7062	−0.5774	FAILED
TARGET−10−PAPGYC−09A−01R	−0.1036	0.0622	PASSED
TARGET−10−PAPHGD−09A−01R	−0.1098	0.054	PASSED
TARGET−10−PANKAK−09A−01R	−0.2551	−0.1108	FAILED
TARGET−10−PANWEZ−09B−01R	−0.0893	0.0218	PASSED
TARGET−10−PAPJHR−09A−01R	−0.6477	−0.4979	FAILED
TARGET−10−PANWYH−09B−01R	0.6076	0.7532	FAILED
TARGET−10−PAMXSP−09A−01R	−0.4896	−0.3431	FAILED
TARGET−10−PASMGZ−09B−01R	0.2473	0.3629	FAILED
TARGET−10−PANWYH−09A−02R	0.2622	0.3756	FAILED
TARGET−10−PARFLV−09A−01R	−1.3934	−1.2104	FAILED
TARGET−10−PARFTR−09A−01R	−0.2019	−0.0635	FAILED
TARGET−10−PARPNM−09A−01R	−0.9854	−0.8391	FAILED
TARGET−10−PAPJHB−09A−01R	−0.0301	0.1367	PASSED
TARGET−10−PAMXHU−09A−01R	−0.2718	−0.1346	FAILED
TARGET−10−PAPBCD−09A−01R	−0.8909	−0.6306	FAILED
TARGET−10−PANWHW−09A−01R	−0.1241	0.0122	PASSED
TARGET−10−PAPBFN−09A−01R	−0.3186	−0.1739	FAILED
TARGET−10−PAPNNX−09A−02R	0.2866	0.4246	FAILED

Exploratory PCA

Use this plot to see if samples are clustered according to the experimental design.

Use ARSyNseq function to correct potential batch effects.

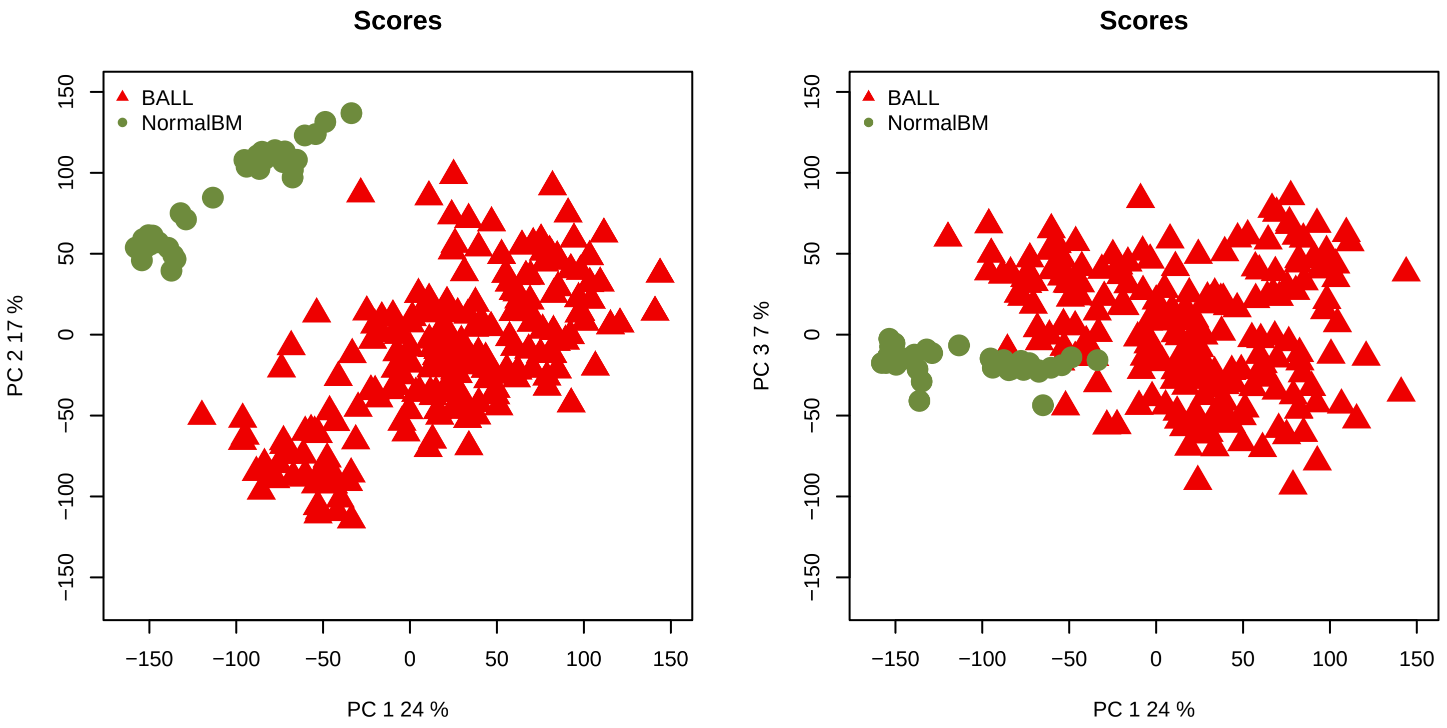

Quality Control of Expression Data
BALL After Normalization

Generated by NOISeq on 22 Nov 2022, 00:08:24

Content

Plot	*Description*
**Biotype detection**	Biotype abundance in the genome with %genes detected (counts > 0) in the sample/condition.<br>Biotype abundance within the sample/condition.
**Biotype expression**	Distribution of gene counts per million per biotype in sample/condition (only genes with counts > 0).
**Saturation**	Number of detected genes (counts > 0) per sample across different sequencing depths
**Expression boxplot**	Distribution of gene counts per million (all biotypes) in each sample/condition
**Expression barplot**	Percentage of genes with >0, >1, >2, >5 or >10 counts per million in each sample/condition.
**Length bias**	Mean gene expression per each length bin. Fitted curve and diagnostic test.
**GC content bias**	Mean gene expression per each GC content bin. Fitted curve and diagnostic test.
**RNA composition bias**	Density plots of log fold changes (M) between pairs of samples.<br>Confidence intervals for the median of M values.
**Exploratory PCA**	Principal Component Analysis score plots for PC1 vs PC2, and PC1 vs PC3.

Biotype detection

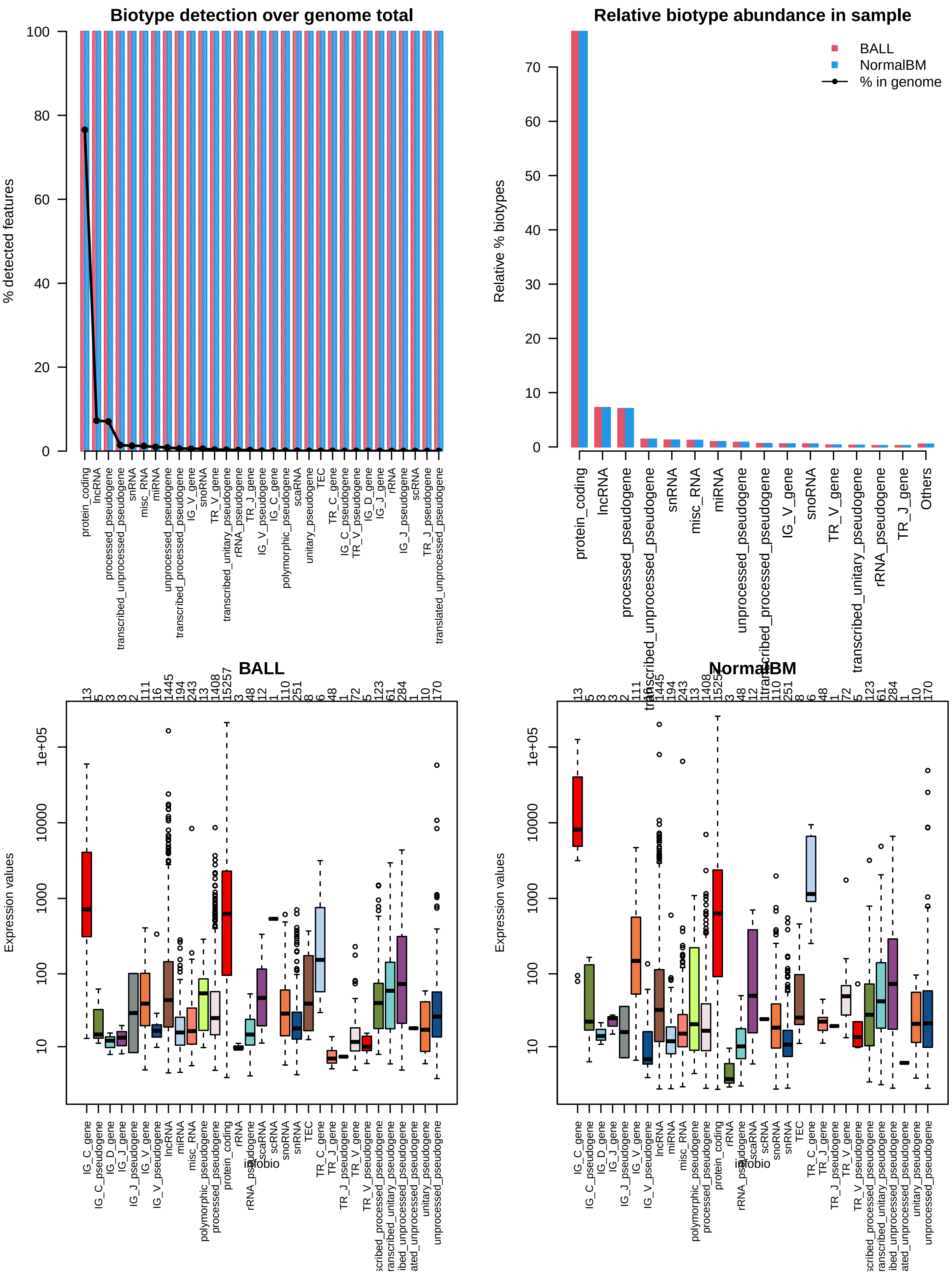

Sequencing depth & Expression quantification

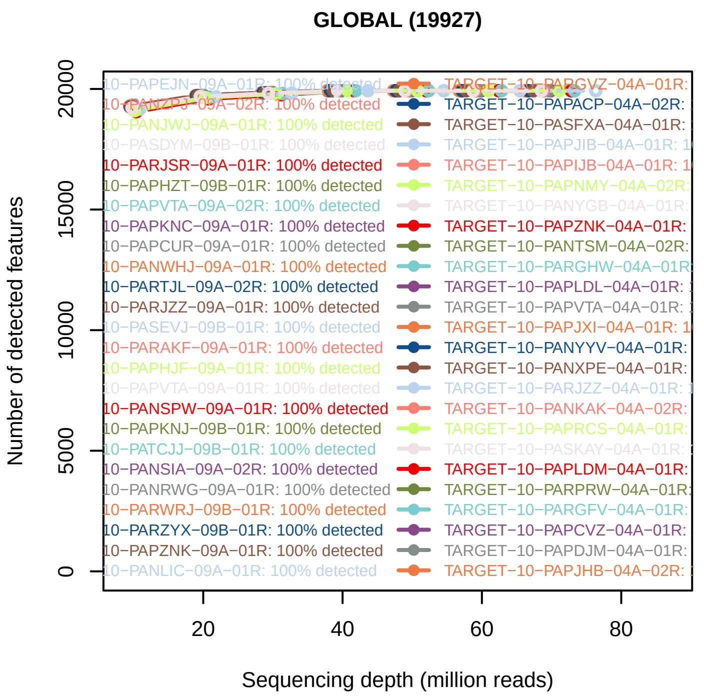

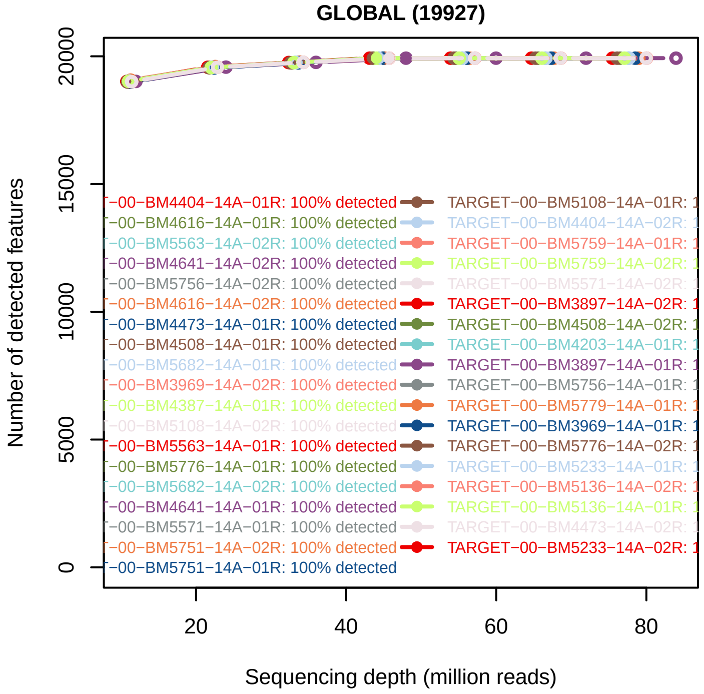

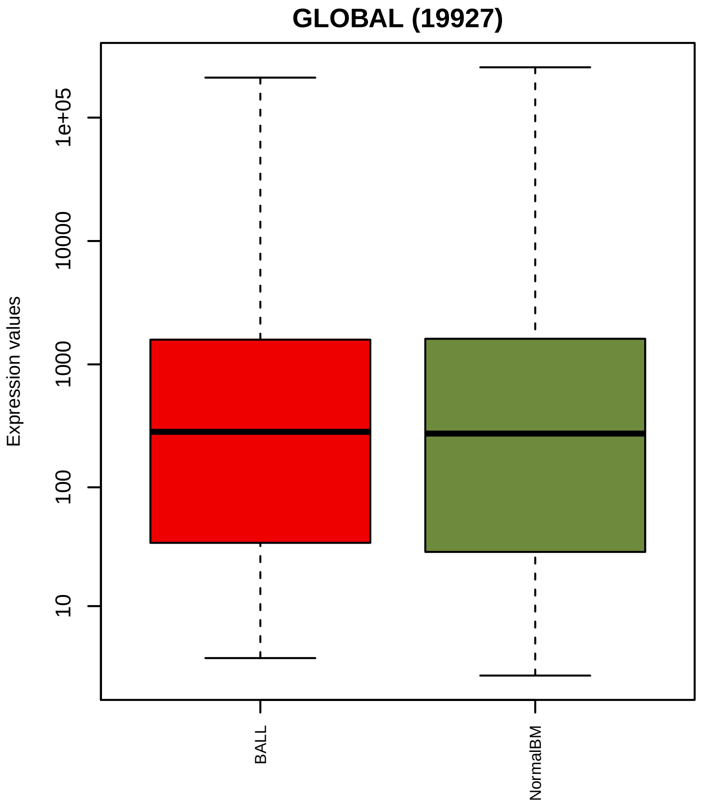

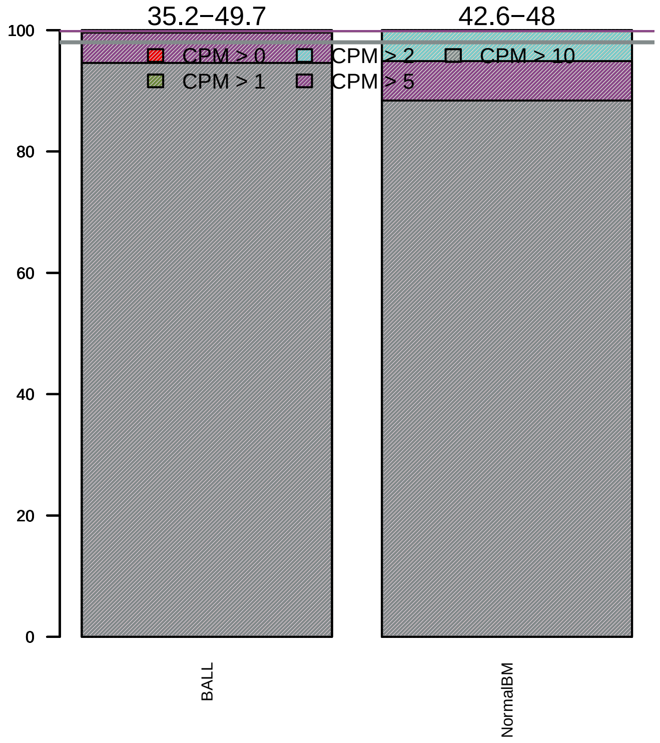

Sequencing bias detection

Diagnostic plot for feature length bias

FAILED. At least one of the model p−values was lower than 0.05 and R2 > 70%.

Normalization for correcting length bias is recommended.

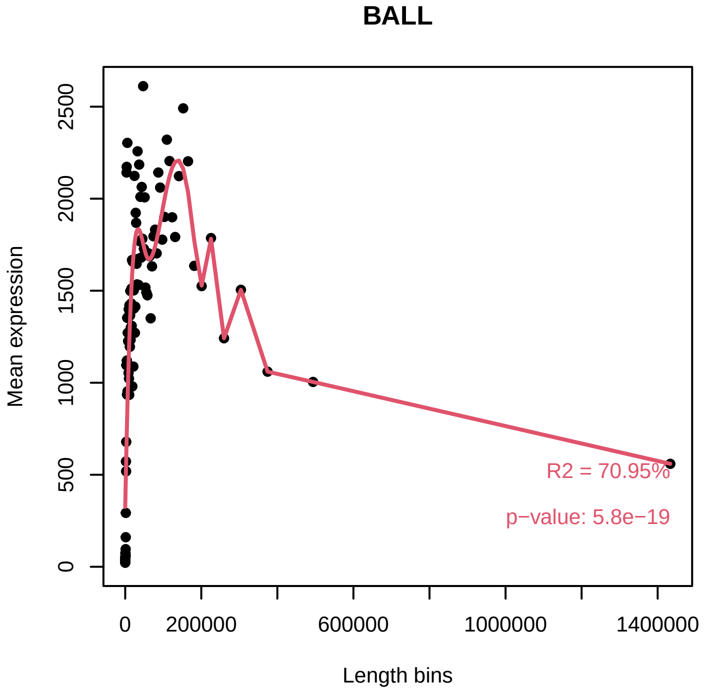

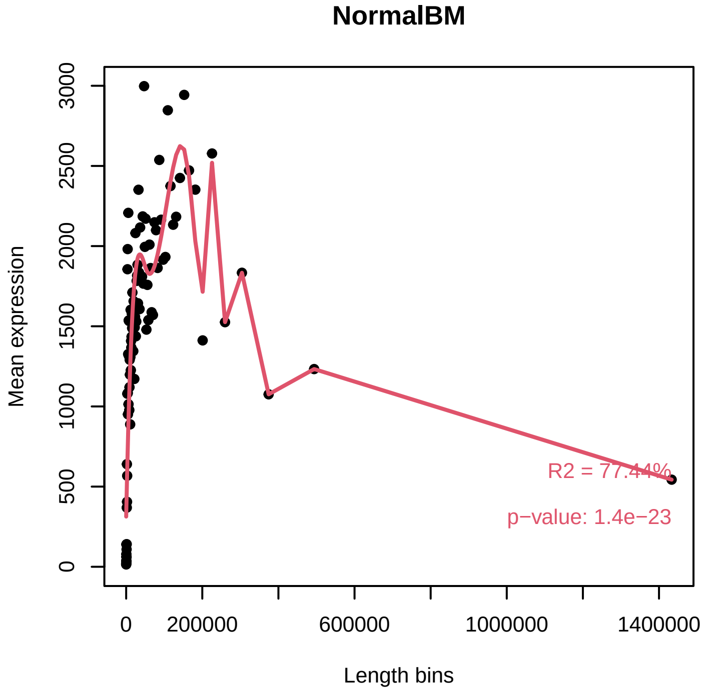

Diagnostic plot for GC content bias

WARNING. At least one of the model p−values was lower than 0.05, but R2 < 70% for at least one condition.

Normalization for correcting GC content bias could be advisable.
Plese check in the plots below the strength of the relationship between GC content and expression.

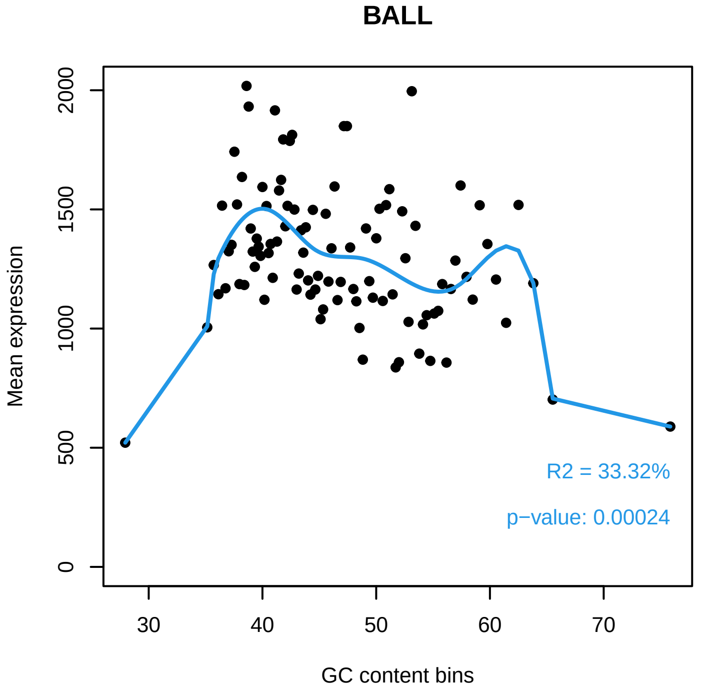

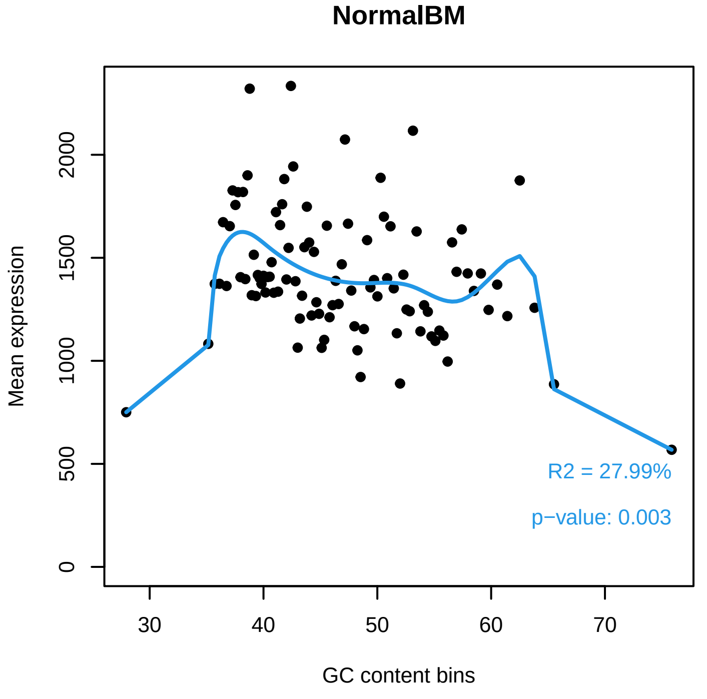

Diagnostic plot for differences in RNA composition

FAILED. There is a pair of samples with significantly different RNA composition

Normalization for correcting this bias is required.

Reference sample: TARGET−10−PARBVI−09A−02R

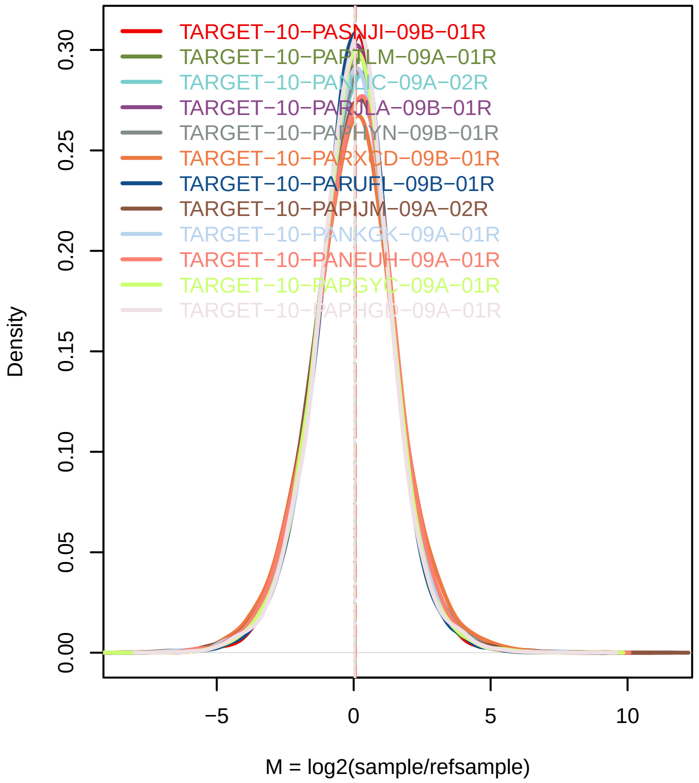

Confidence intervals for median of M values

Sample	0.01%	99.99%	Diagnostic Test
TARGET−10−PASNJI−09B−01R	0.013	0.0856	FAILED
TARGET−10−PAPTLM−09A−01R	0.0183	0.0961	FAILED
TARGET−10−PANLIC−09A−02R	0.0085	0.0899	FAILED
TARGET−10−PARJLA−09B−01R	0.0108	0.0831	FAILED
TARGET−10−PAPHYN−09B−01R	0.0223	0.1046	FAILED
TARGET−10−PARXCD−09B−01R	0.0013	0.0942	FAILED
TARGET−10−PARUFL−09B−01R	0.0084	0.0784	FAILED
TARGET−10−PAPIJM−09A−02R	−0.0149	0.0639	PASSED
TARGET−10−PANKGK−09A−01R	1e−04	0.0826	FAILED
TARGET−10−PANEUH−09A−01R	0.0491	0.1136	FAILED
TARGET−10−PAPGYC−09A−01R	−0.0025	0.0755	PASSED
TARGET−10−PAPHGD−09A−01R	0.0127	0.0876	FAILED
TARGET−10−PANKAK−09A−01R	0.0343	0.1039	FAILED
TARGET−10−PANWEZ−09B−01R	0.0161	0.0967	FAILED
TARGET−10−PAPJHR−09A−01R	0.0257	0.0998	FAILED
TARGET−10−PANWYH−09B−01R	0.0085	0.0831	FAILED
TARGET−10−PAMXSP−09A−01R	0.0155	0.1002	FAILED
TARGET−10−PASMGZ−09B−01R	0.0157	0.088	FAILED
TARGET−10−PANWYH−09A−02R	0.02	0.0935	FAILED
TARGET−10−PARFLV−09A−01R	0.0194	0.0896	FAILED
TARGET−10−PARFTR−09A−01R	0.031	0.1092	FAILED
TARGET−10−PARPNM−09A−01R	0.0057	0.0734	FAILED
TARGET−10−PAPJHB−09A−01R	4e−04	0.0668	FAILED
TARGET−10−PAMXHJ−09A−01R	0.0223	0.1029	FAILED
TARGET−10−PAPBCI−09A−01R	0	0.0841	PASSED
TARGET−10−PANWHW−09A−01R	0.0019	0.0994	FAILED
TARGET−10−PAPBFN−09A−01R	0.0054	0.0897	FAILED
TARGET−10−PAPNNX−09A−02R	5e−04	0.0771	FAILED

Exploratory PCA

Use this plot to see if samples are clustered according to the experimental design.

Use ARSyNseq function to correct potential batch effects.

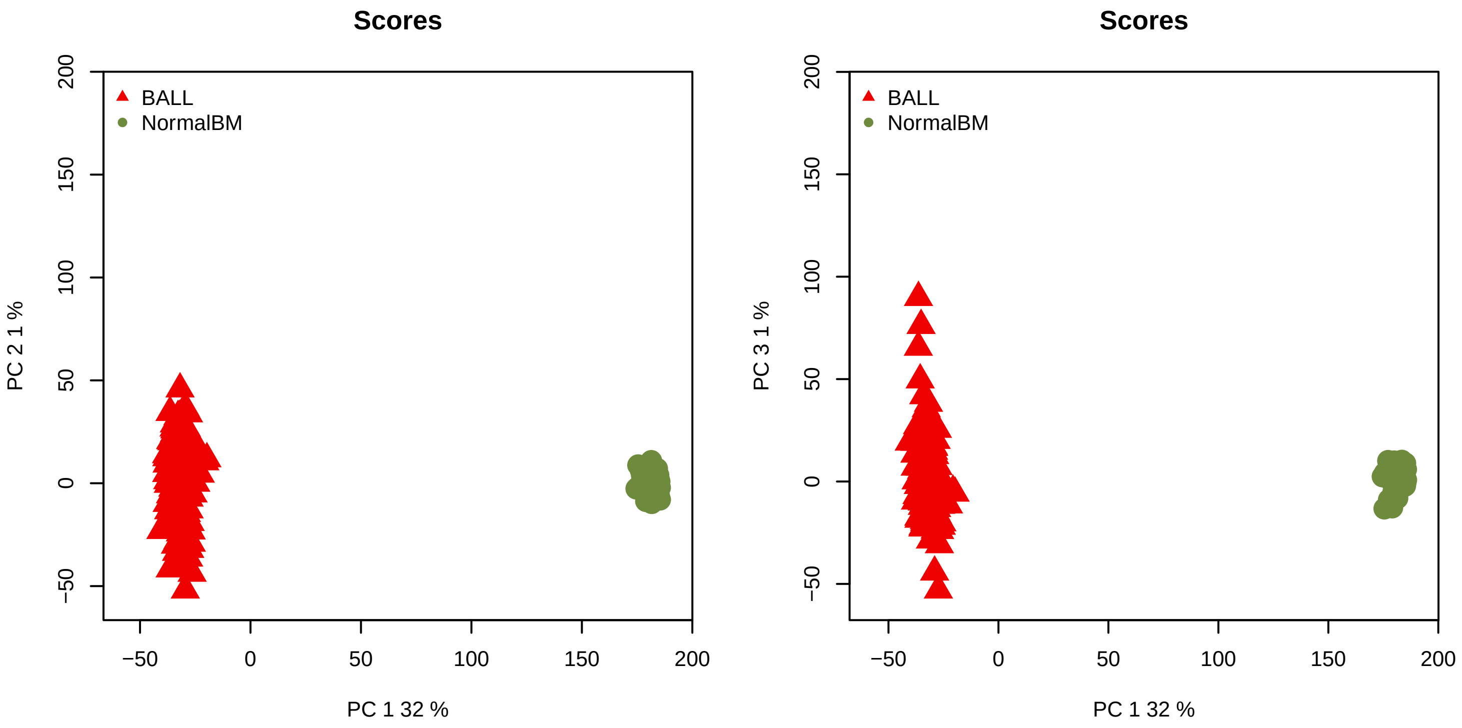

Quality Control of Expression Data
MM Before Normalization

Generated by NOISeq on 22 Nov 2022, 00:05:26

Content

Plot	*Description*
**Biotype detection**	Biotype abundance in the genome with %genes detected (counts > 0) in the sample/condition.<br>Biotype abundance within the sample/condition.
**Biotype expression**	Distribution of gene counts per million per biotype in sample/condition (only genes with counts > 0).
**Saturation**	Number of detected genes (counts > 0) per sample across different sequencing depths
**Expression boxplot**	Distribution of gene counts per million (all biotypes) in each sample/condition
**Expression barplot**	Percentage of genes with >0, >1, >2, >5 or >10 counts per million in each sample/condition.
**Length bias**	Mean gene expression per each length bin. Fitted curve and diagnostic test.
**GC content bias**	Mean gene expression per each GC content bin. Fitted curve and diagnostic test.
**RNA composition bias**	Density plots of log fold changes (M) between pairs of samples.<br>Confidence intervals for the median of M values.
**Exploratory PCA**	Principal Component Analysis score plots for PC1 vs PC2, and PC1 vs PC3.

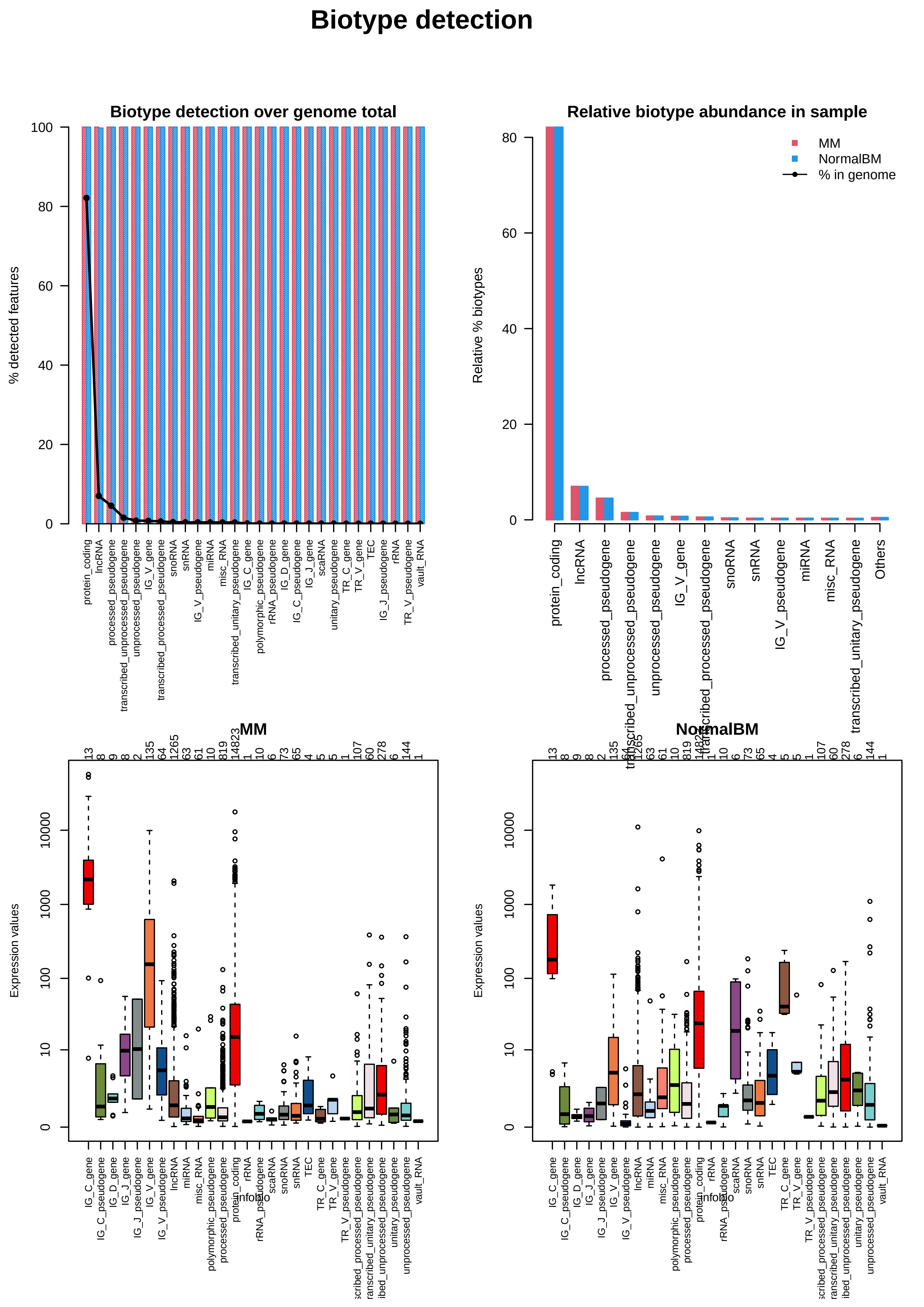
Biotype detection
Biotype detection over genome total
% detected features
protein_coding
lncRNA
processed_pseudogene
transcribed_unprocessed_pseudogene
unprocessed_pseudogene
IG_V_gene
transcribed_processed_pseudogene
snoRNA
snRNA
IG_V_pseudogene
miRNA
misc_RNA
transcribed_unitary_pseudogene
IG_C_gene
polymorphic_pseudogene
rRNA_pseudogene
IG_D_gene
IG_C_pseudogene
IG_J_gene
scaRNA
unitary_pseudogene
TR_C_gene
TR_V_gene
TEC
IG_J_pseudogene
rRNA
TR_V_pseudogene
vault_RNA
Relative biotype abundance in sample
Relative % biotypes
MM
NormalBM
% in genome
Others
MM
NormalBM
Expression values
infobio

Sequencing depth & Expression quantification

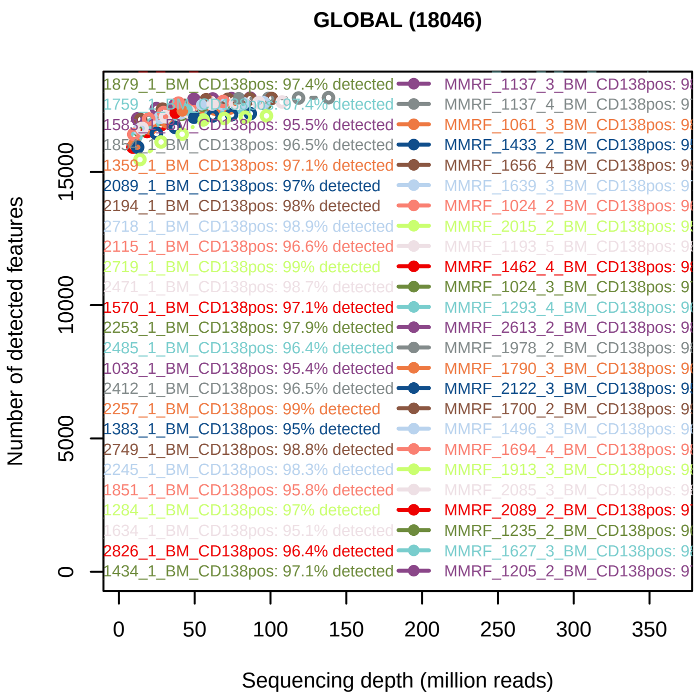

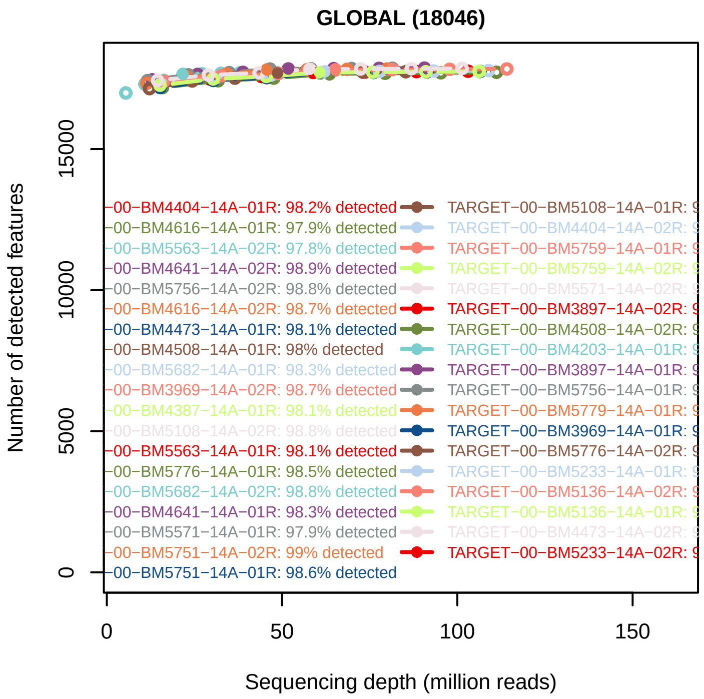

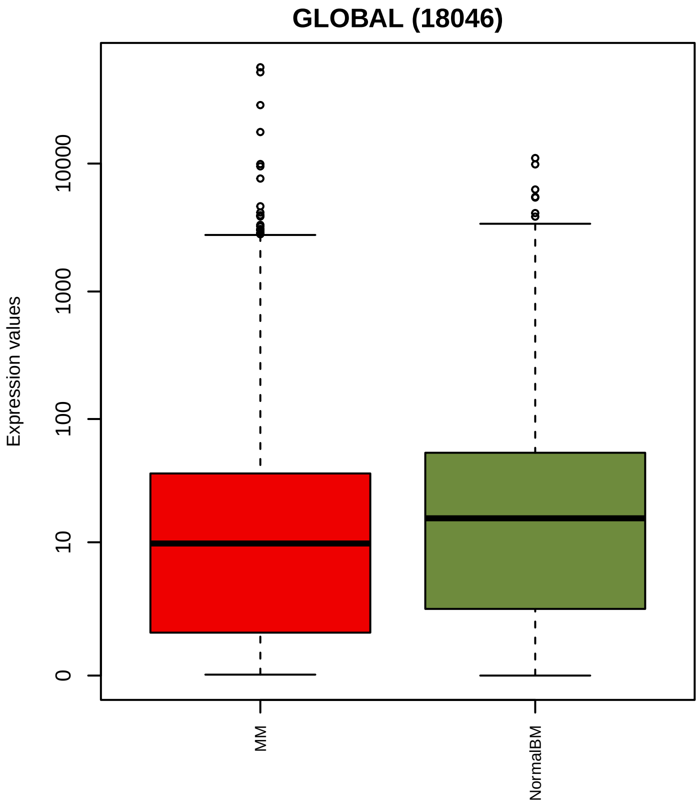

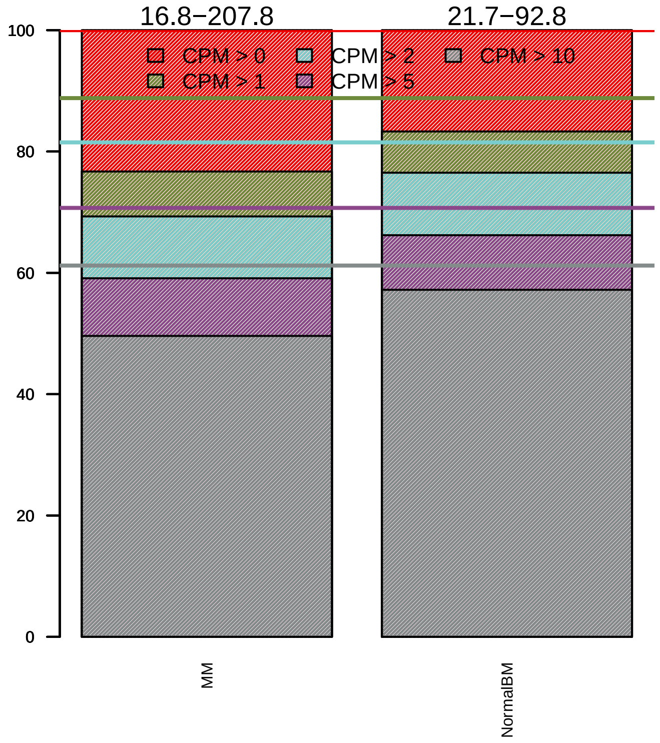

Sequencing bias detection

Diagnostic plot for feature length bias

WARNING. At least one of the model p−values was lower than 0.05, but R2 < 70% for at least one condition.

Normalization for correcting length bias could be advisable.
Plese check in the plots below the strength of the relationship between length and expression.

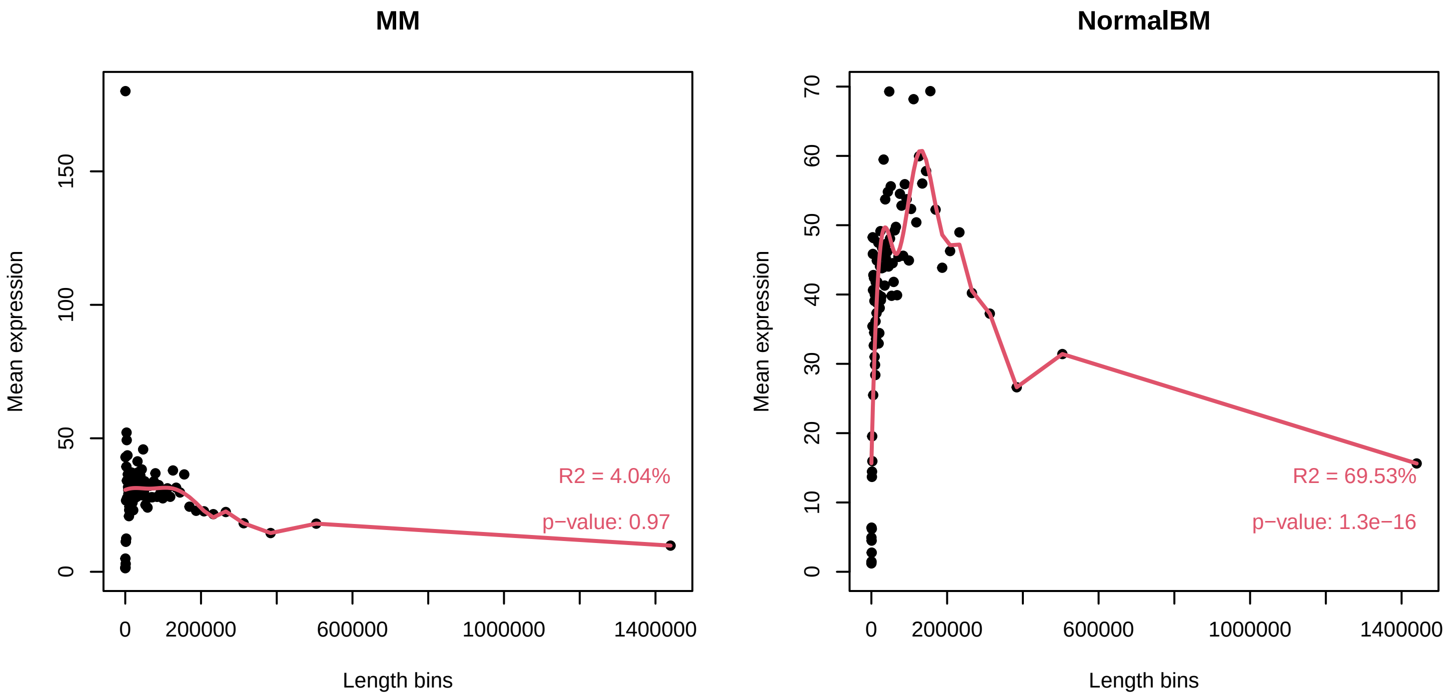

Diagnostic plot for GC content bias

WARNING. At least one of the model p−values was lower than 0.05, but R2 < 70% for at least one condition.

Normalization for correcting GC content bias could be advisable.
Plese check in the plots below the strength of the relationship between GC content and expression.

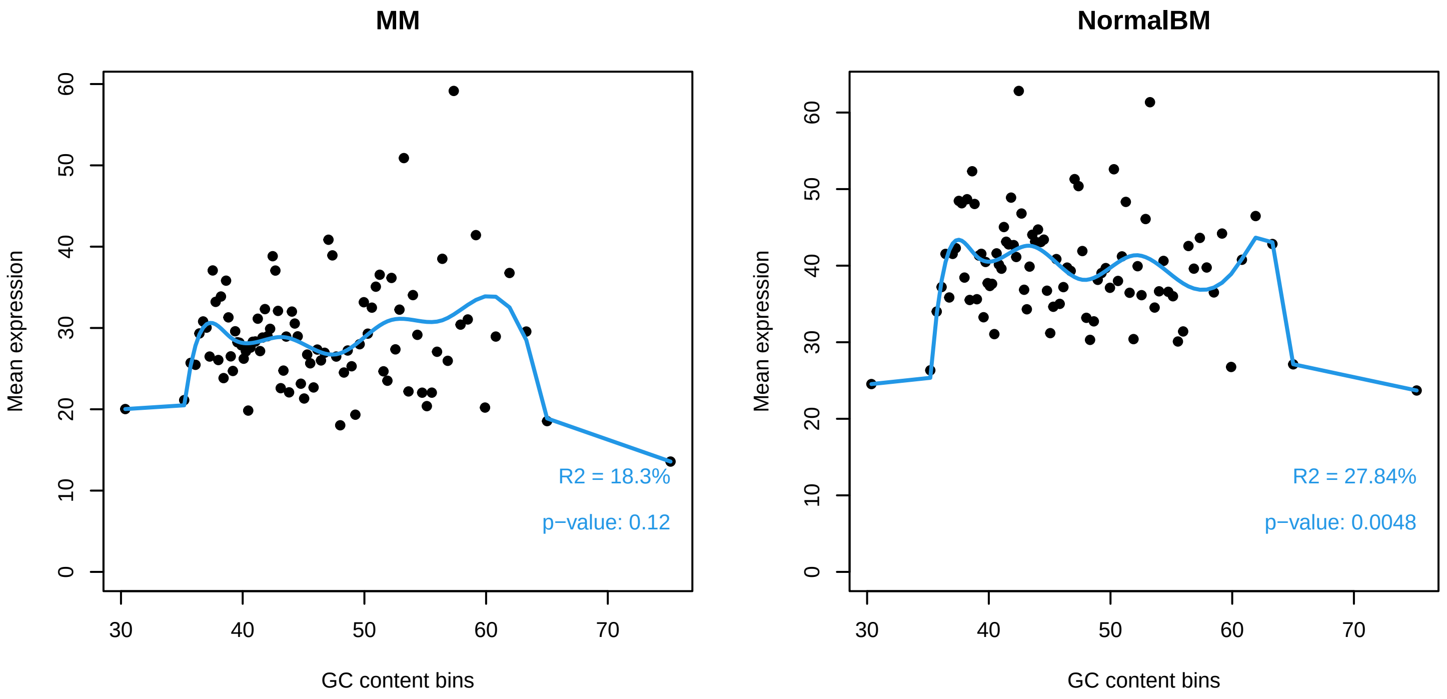

Diagnostic plot for differences in RNA composition

FAILED. There is a pair of samples with significantly different RNA composition

Normalization for correcting this bias is required.

Reference sample: MMRF_2173_1_BM_CD138pos

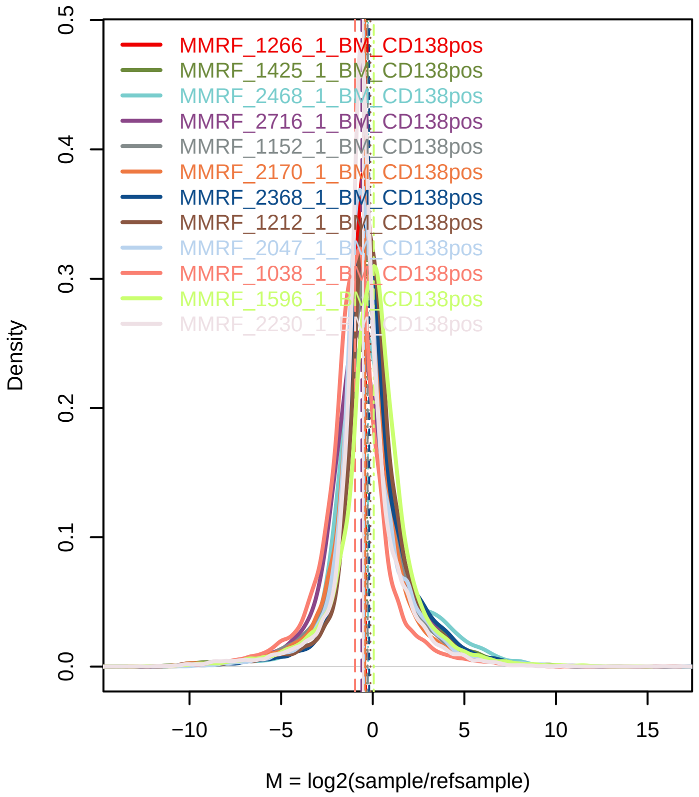

Confidence intervals for median of M values

Sample	0%	100%	Diagnostic Test
MMRF_1266_1_BM_CD138pos	−0.4057	−0.3366	FAILED
MMRF_1425_1_BM_CD138pos	−0.3642	−0.2748	FAILED
MMRF_2468_1_BM_CD138pos	−0.3087	−0.2185	FAILED
MMRF_2716_1_BM_CD138pos	−0.6702	−0.5725	FAILED
MMRF_1152_1_BM_CD138pos	−0.3222	−0.2395	FAILED
MMRF_2170_1_BM_CD138pos	−0.441	−0.4134	FAILED
MMRF_2368_1_BM_CD138pos	−0.2198	−0.1633	FAILED
MMRF_1212_1_BM_CD138pos	−0.1636	−0.0953	FAILED
MMRF_2047_1_BM_CD138pos	−0.5059	−0.4415	FAILED
MMRF_1038_1_BM_CD138pos	−0.9959	−0.9349	FAILED
MMRF_1596_1_BM_CD138pos	−0.0087	0.0897	PASSED
MMRF_2230_1_BM_CD138pos	−0.557	−0.5076	FAILED
MMRF_2480_1_BM_CD138pos	−0.1531	−0.0823	FAILED
MMRF_2455_1_BM_CD138pos	0.0368	0.0979	FAILED
MMRF_1490_1_BM_CD138pos	−0.1988	−0.127	FAILED
MMRF_1179_1_BM_CD138pos	−0.3196	−0.2427	FAILED
MMRF_1252_1_BM_CD138pos	−0.3978	−0.3288	FAILED
MMRF_1364_1_BM_CD138pos	0.1172	0.2191	FAILED
MMRF_2699_1_BM_CD138pos	−0.318	−0.2234	FAILED
MMRF_2751_1_BM_CD138pos	−0.2504	−0.1957	FAILED
MMRF_2543_1_BM_CD138pos	−0.5404	−0.4784	FAILED
MMRF_2487_1_BM_CD138pos	−0.3449	−0.274	FAILED
MMRF_2267_1_BM_CD138pos	−0.2417	−0.1883	FAILED
MMRF_1932_1_BM_CD138pos	0.2768	0.3532	FAILED
MMRF_2082_1_BM_CD138pos	−0.5388	−0.4824	FAILED
MMRF_1613_1_BM_CD138pos	−0.3112	−0.2438	FAILED
MMRF_2062_1_BM_CD138pos	0.204	0.2784	FAILED
MMRF_2384_1_BM_CD138pos	−0.2321	−0.1587	FAILED

Exploratory PCA

Use this plot to see if samples are clustered according to the experimental design.

Use ARSyNseq function to correct potential batch effects.

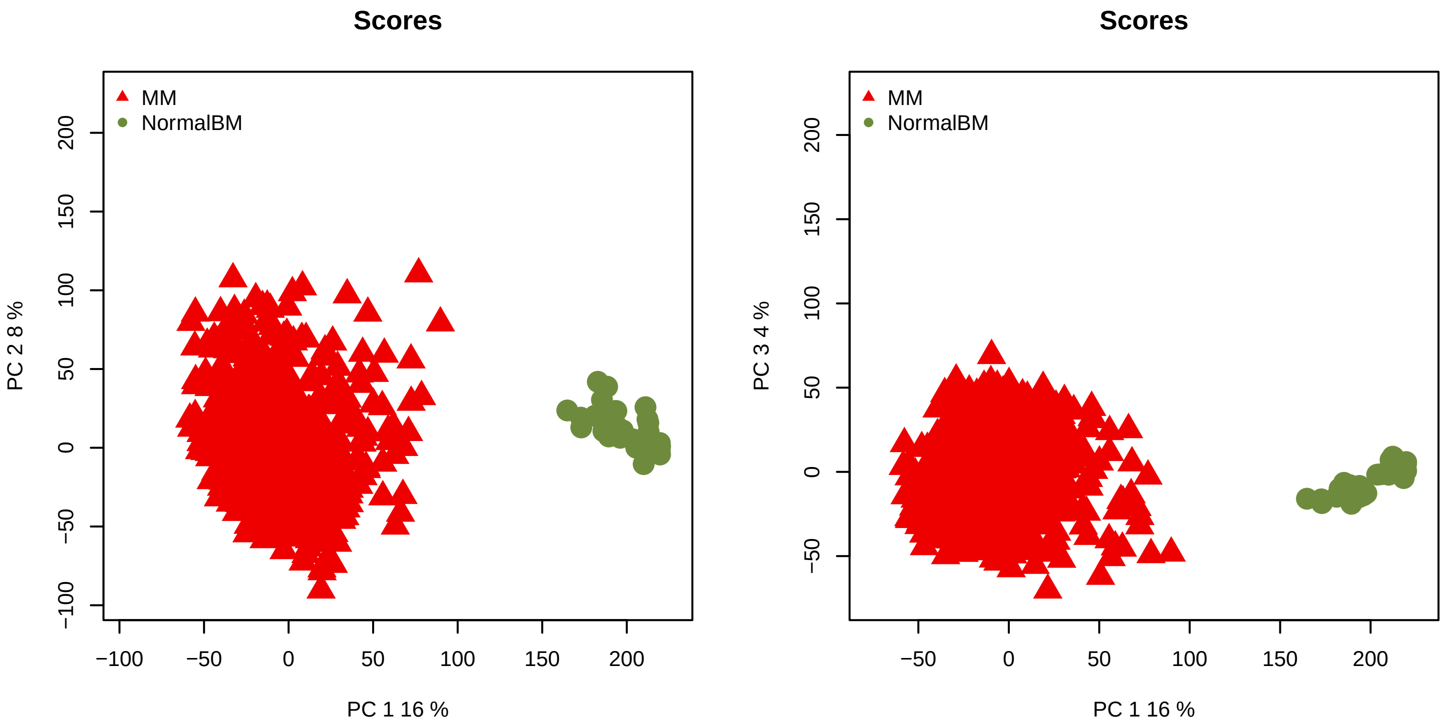

Quality Control of Expression Data
MM After Normalization

Generated by NOISeq on 22 Nov 2022, 00:32:07

Content

Plot	*Description*
**Biotype detection**	Biotype abundance in the genome with %genes detected (counts > 0) in the sample/condition.<br>Biotype abundance within the sample/condition.
**Biotype expression**	Distribution of gene counts per million per biotype in sample/condition (only genes with counts > 0).
**Saturation**	Number of detected genes (counts > 0) per sample across different sequencing depths
**Expression boxplot**	Distribution of gene counts per million (all biotypes) in each sample/condition
**Expression barplot**	Percentage of genes with >0, >1, >2, >5 or >10 counts per million in each sample/condition.
**Length bias**	Mean gene expression per each length bin. Fitted curve and diagnostic test.
**GC content bias**	Mean gene expression per each GC content bin. Fitted curve and diagnostic test.
**RNA composition bias**	Density plots of log fold changes (M) between pairs of samples.<br>Confidence intervals for the median of M values.
**Exploratory PCA**	Principal Component Analysis score plots for PC1 vs PC2, and PC1 vs PC3.

Biotype detection

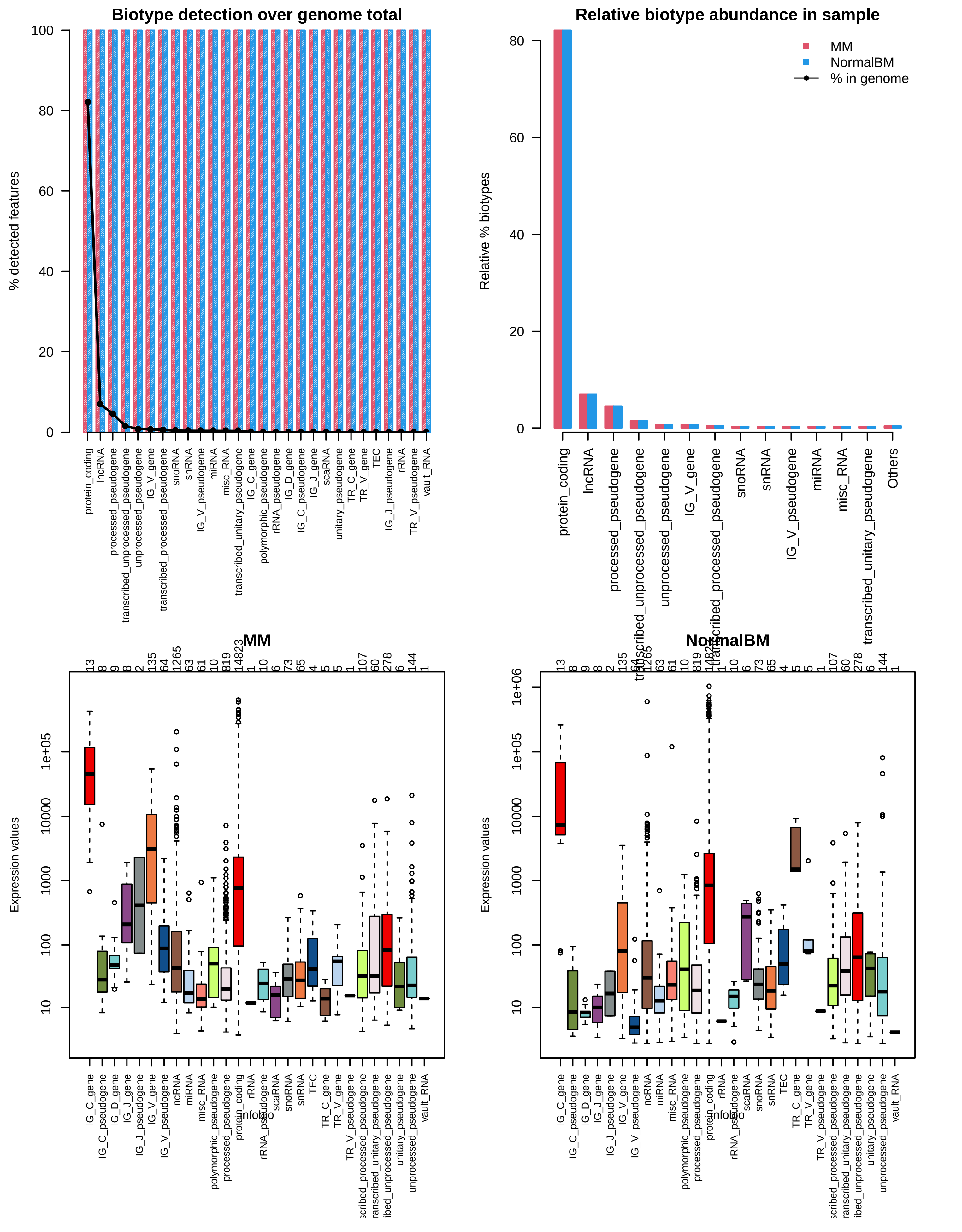

Sequencing depth & Expression quantification

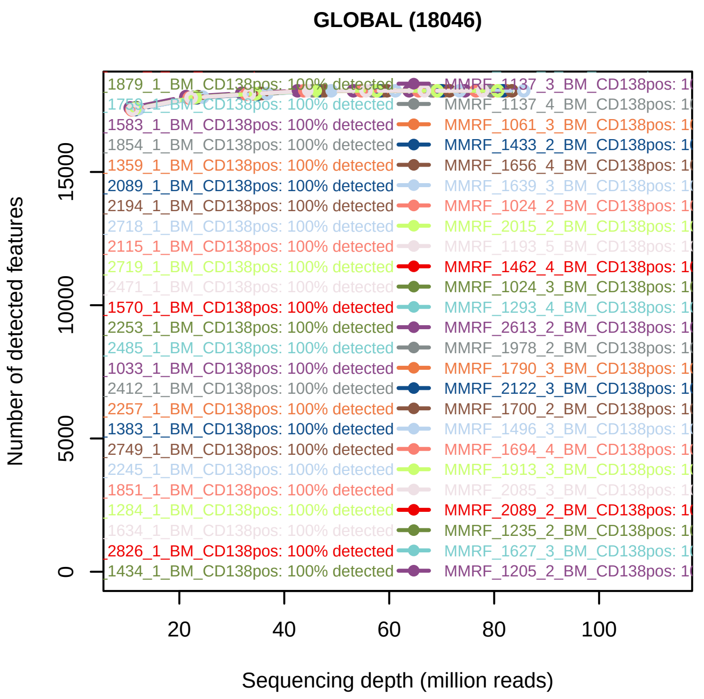

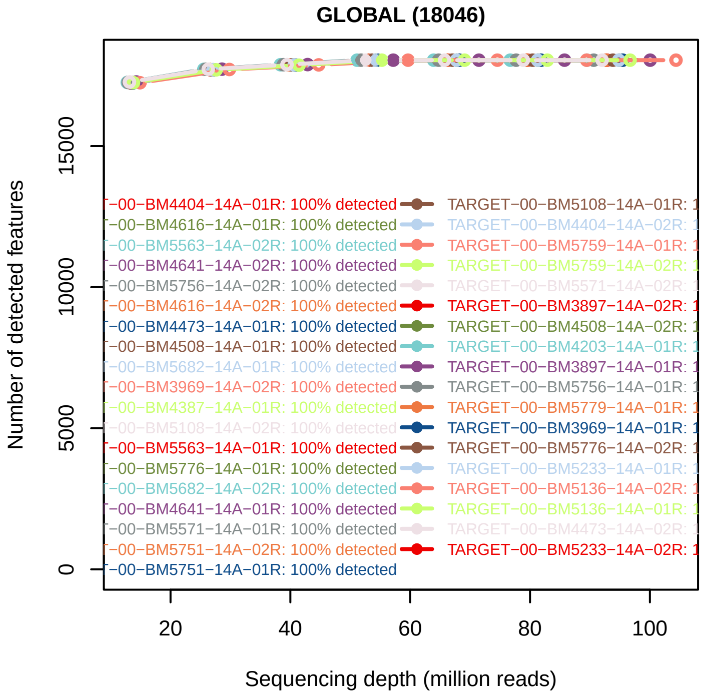

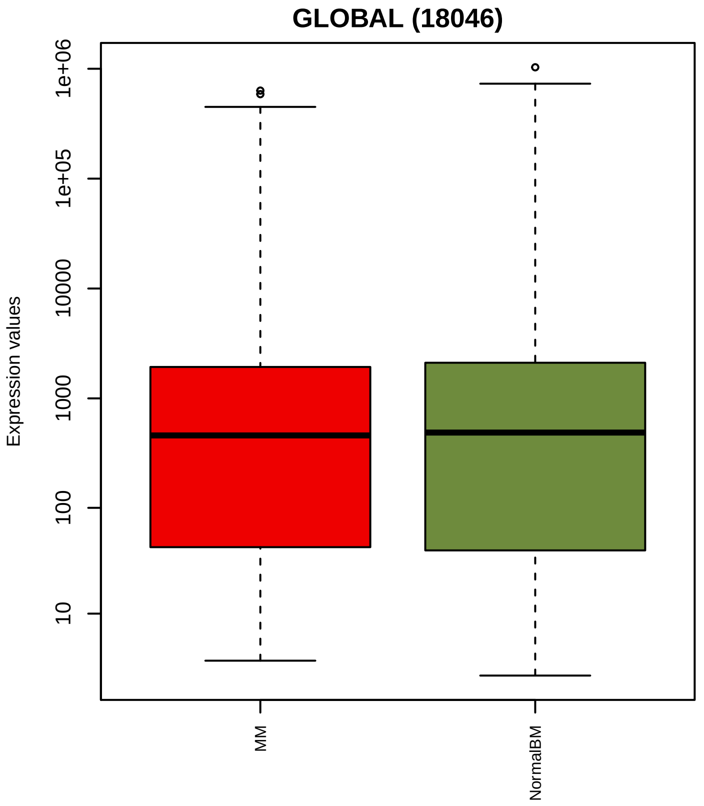

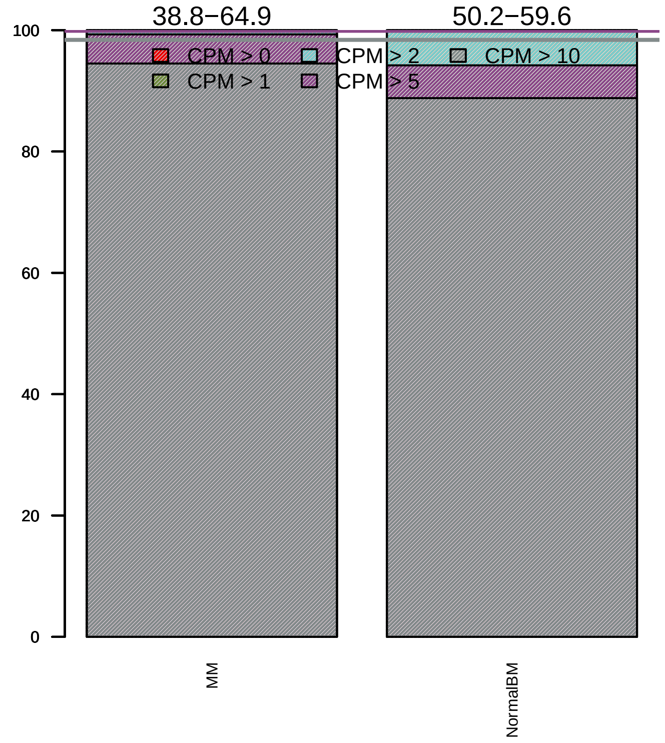

Sequencing bias detection

Diagnostic plot for feature length bias

WARNING. At least one of the model p−values was lower than 0.05, but R2 < 70% for at least one condition.

Normalization for correcting length bias could be advisable.
Plese check in the plots below the strength of the relationship between length and expression.

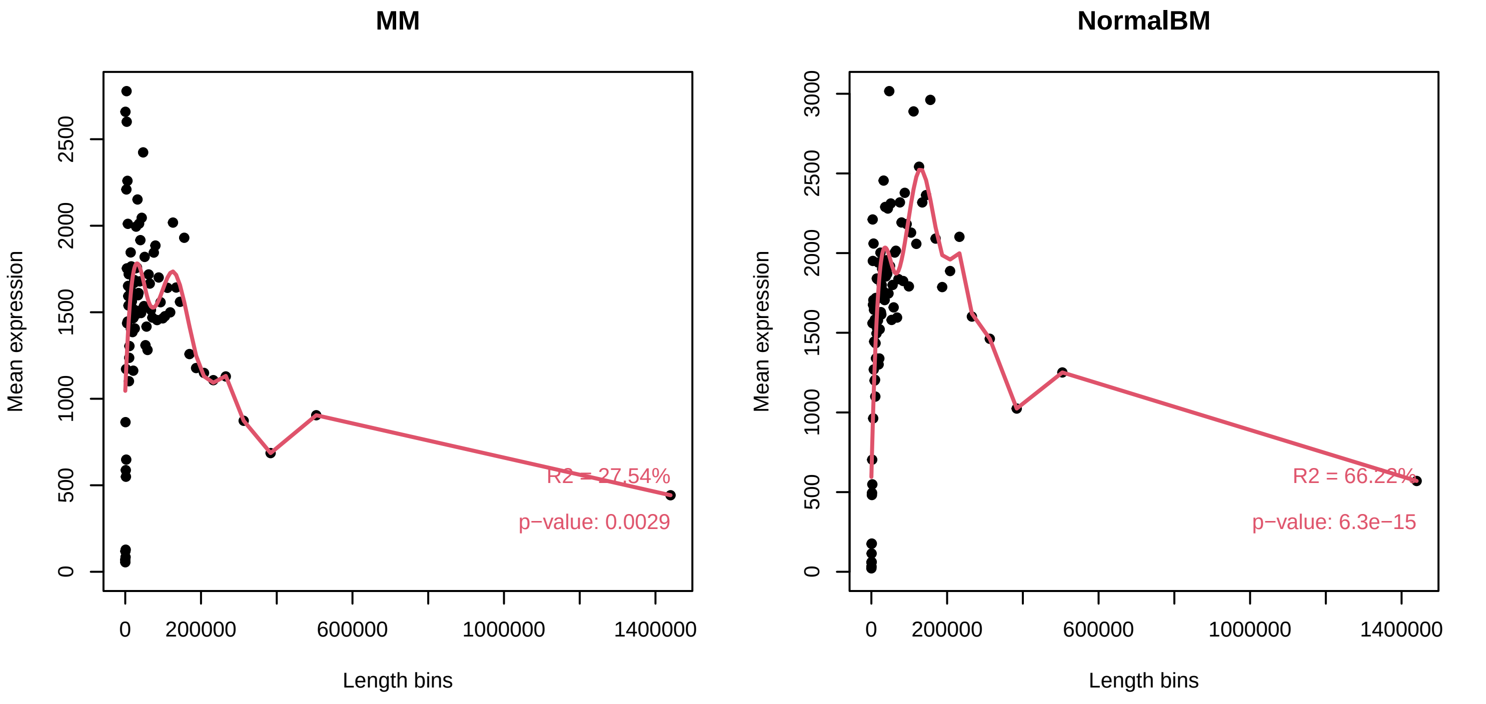

Diagnostic plot for GC content bias

WARNING. At least one of the model p−values was lower than 0.05, but R2 < 70% for at least one condition.

Normalization for correcting GC content bias could be advisable.
Plese check in the plots below the strength of the relationship between GC content and expression.

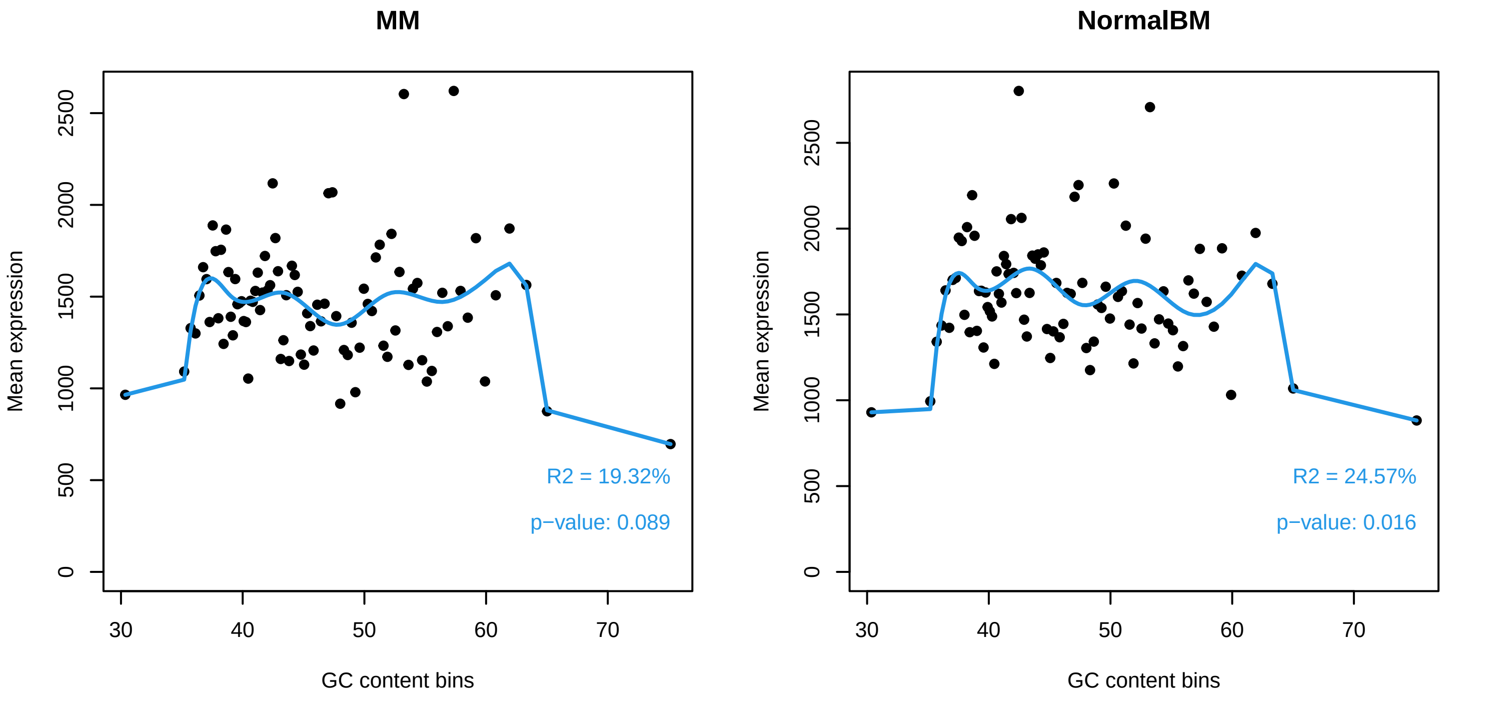

Diagnostic plot for differences in RNA composition

FAILED. There is a pair of samples with significantly different RNA composition

Normalization for correcting this bias is required.

Reference sample: MMRF_2173_1_BM_CD138pos

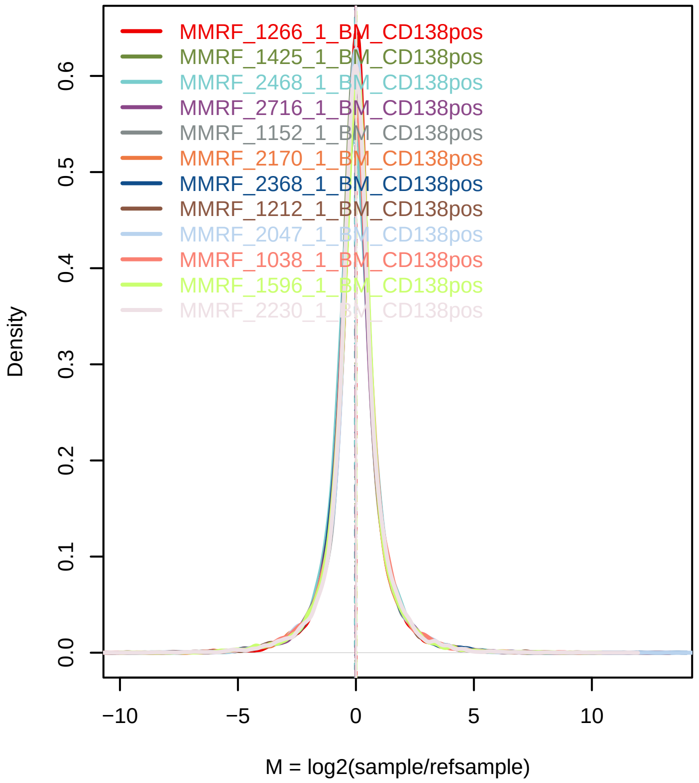

Confidence intervals for median of M values

Sample	0%	100%	Diagnostic Test
MMRF_1266_1_BM_CD138pos	[illegible]	0.0385	FAILED
MMRF_1425_1_BM_CD138pos	[illegible]	0.0042	PASSED
MMRF_2468_1_BM_CD138pos	[illegible]	−0.0243	FAILED
MMRF_2716_1_BM_CD138pos	[illegible]	−0.0022	FAILED
MMRF_1152_1_BM_CD138pos	[illegible]	0.0186	PASSED
MMRF_2170_1_BM_CD138pos	[illegible]	0.0286	PASSED
MMRF_2368_1_BM_CD138pos	[illegible]	0.024	PASSED
MMRF_1212_1_BM_CD138pos	[illegible]	0.031	PASSED
MMRF_2047_1_BM_CD138pos	[illegible]	0.034	PASSED
MMRF_1038_1_BM_CD138pos	[illegible]	0.024	PASSED
MMRF_1596_1_BM_CD138pos	[illegible]	0.0387	FAILED
MMRF_2230_1_BM_CD138pos	[illegible]	0.0228	PASSED
MMRF_2480_1_BM_CD138pos	[illegible]	0.0134	PASSED
MMRF_2455_1_BM_CD138pos	[illegible]	0.0268	PASSED
MMRF_1490_1_BM_CD138pos	[illegible]	0.0118	PASSED
MMRF_1179_1_BM_CD138pos	[illegible]	0.0061	PASSED
MMRF_1252_1_BM_CD138pos	[illegible]	0.0185	PASSED
MMRF_1364_1_BM_CD138pos	[illegible]	0.0138	PASSED
MMRF_2699_1_BM_CD138pos	[illegible]	0.0241	PASSED
MMRF_2751_1_BM_CD138pos	[illegible]	0.027	PASSED
MMRF_2543_1_BM_CD138pos	[illegible]	0.0174	PASSED
MMRF_2487_1_BM_CD138pos	[illegible]	0.0182	PASSED
MMRF_2267_1_BM_CD138pos	[illegible]	0.0358	FAILED
MMRF_1932_1_BM_CD138pos	[illegible]	0.0236	PASSED
MMRF_2082_1_BM_CD138pos	[illegible]	0.0395	PASSED
MMRF_1613_1_BM_CD138pos	[illegible]	0.0338	PASSED
MMRF_2062_1_BM_CD138pos	[illegible]	0.0345	PASSED
MMRF_2384_1_BM_CD138pos	[illegible]	0.0287	PASSED

Exploratory PCA

Use this plot to see if samples are clustered according to the experimental design.

Use ARSyNseq function to correct potential batch effects.

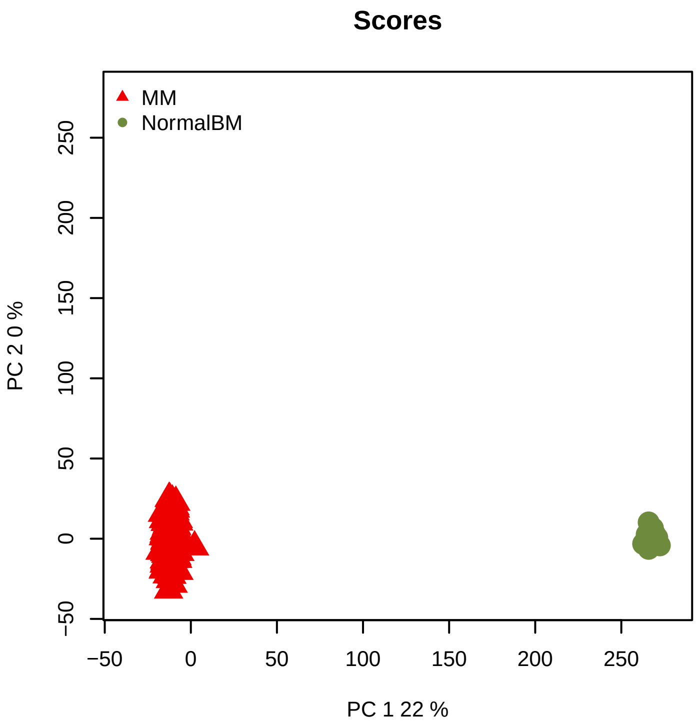

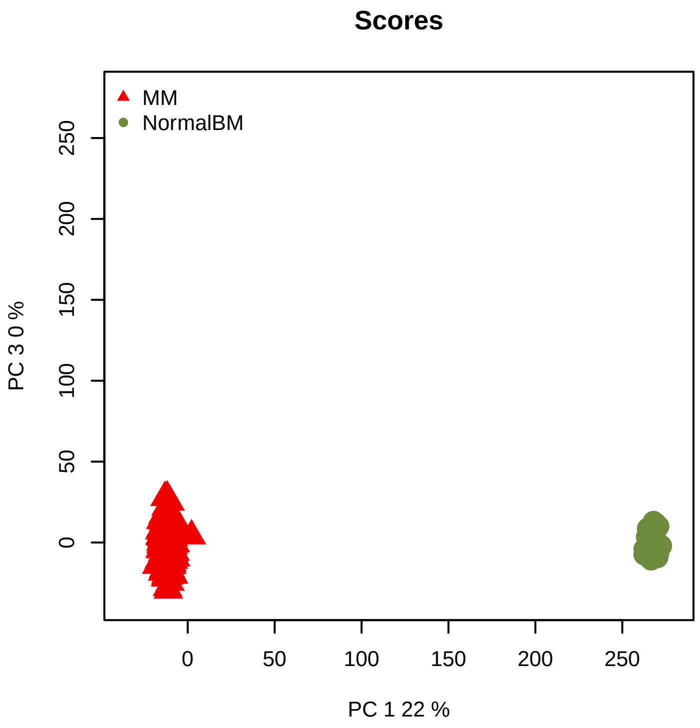

Quality Control of Expression Data
TALL Before Normalization

Generated by NOISeq on 21 Nov 2022, 23:43:01

Content

Plot	*Description*
**Biotype detection**	Biotype abundance in the genome with %genes detected (counts > 0) in the sample/condition.<br>Biotype abundance within the sample/condition.
**Biotype expression**	Distribution of gene counts per million per biotype in sample/condition (only genes with counts > 0).
**Saturation**	Number of detected genes (counts > 0) per sample across different sequencing depths
**Expression boxplot**	Distribution of gene counts per million (all biotypes) in each sample/condition
**Expression barplot**	Percentage of genes with >0, >1, >2, >5 or >10 counts per million in each sample/condition.
**Length bias**	Mean gene expression per each length bin. Fitted curve and diagnostic test.
**GC content bias**	Mean gene expression per each GC content bin. Fitted curve and diagnostic test.
**RNA composition bias**	Density plots of log fold changes (M) between pairs of samples.<br>Confidence intervals for the median of M values.
**Exploratory PCA**	Principal Component Analysis score plots for PC1 vs PC2, and PC1 vs PC3.

Biotype detection

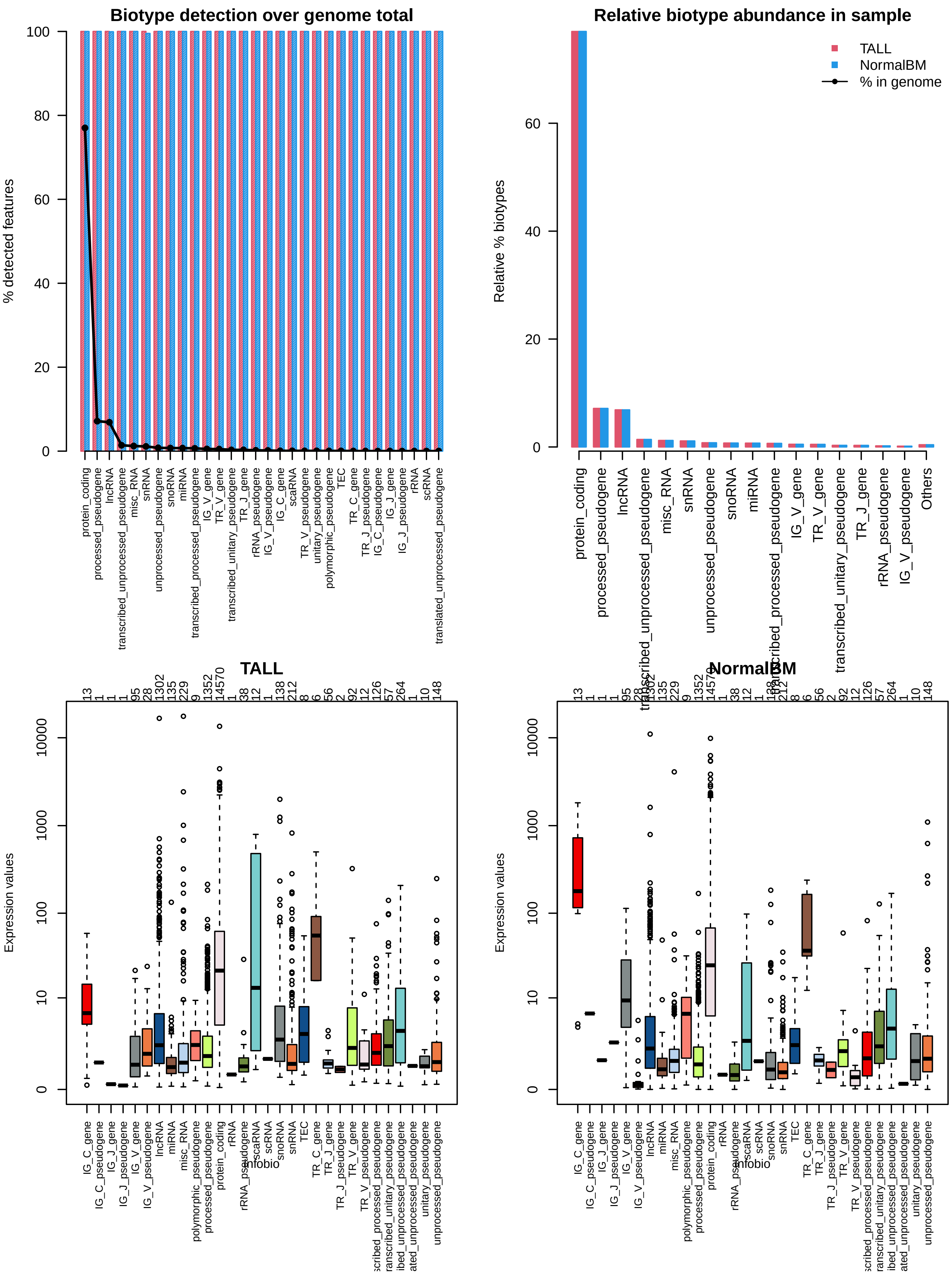

Sequencing depth & Expression quantification

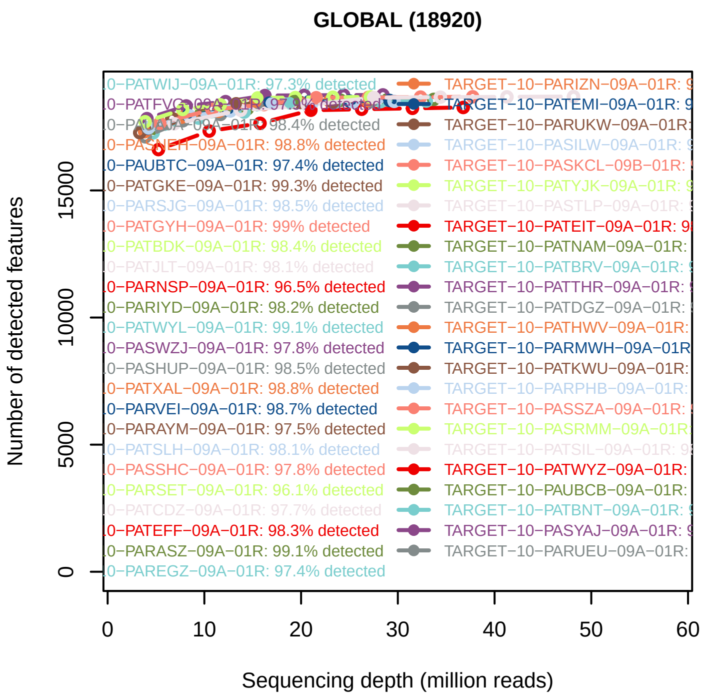

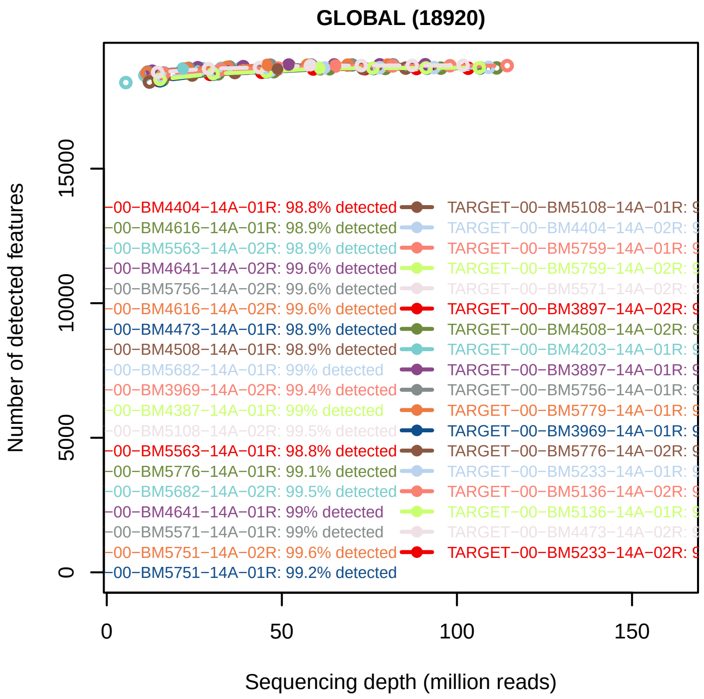

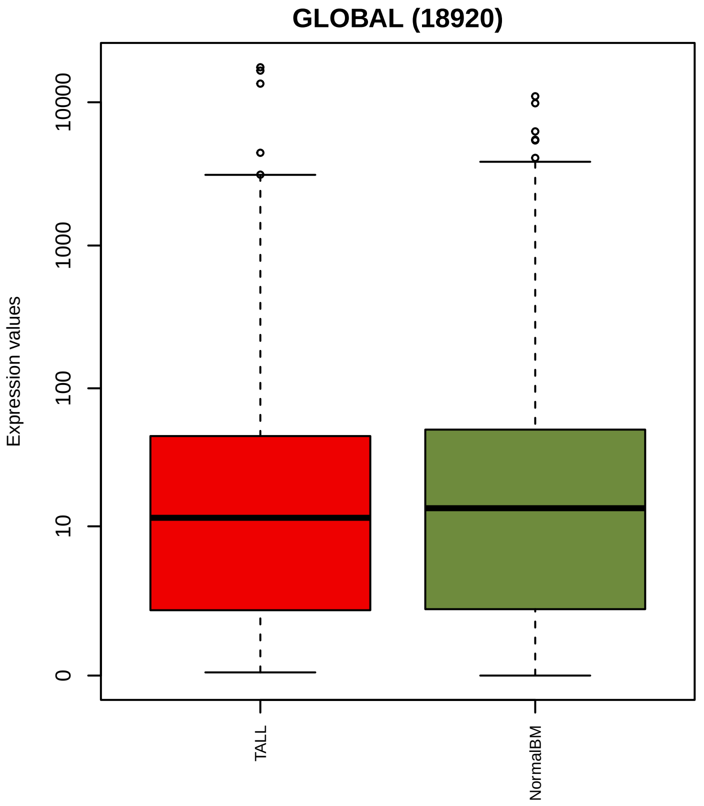

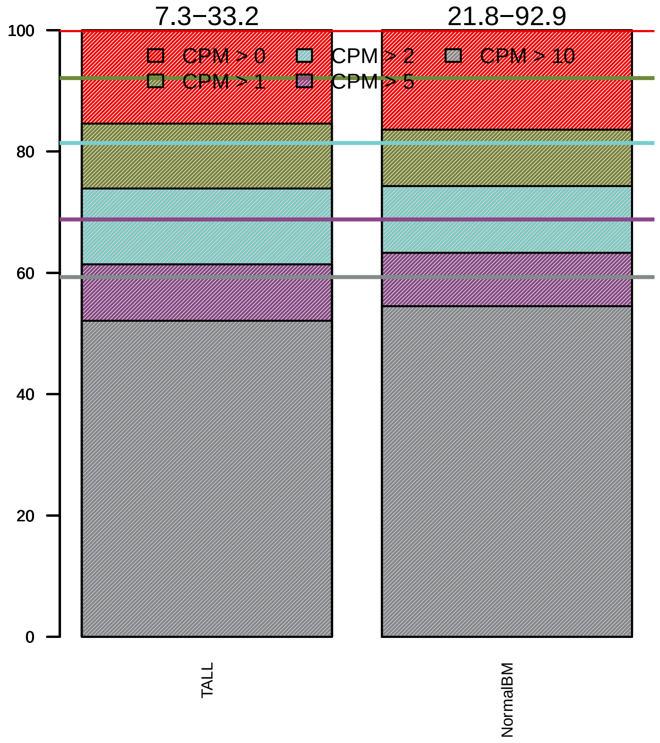

Sequencing bias detection

Diagnostic plot for feature length bias

FAILED. At least one of the model p−values was lower than 0.05 and R2 > 70%.

Normalization for correcting length bias is recommended.

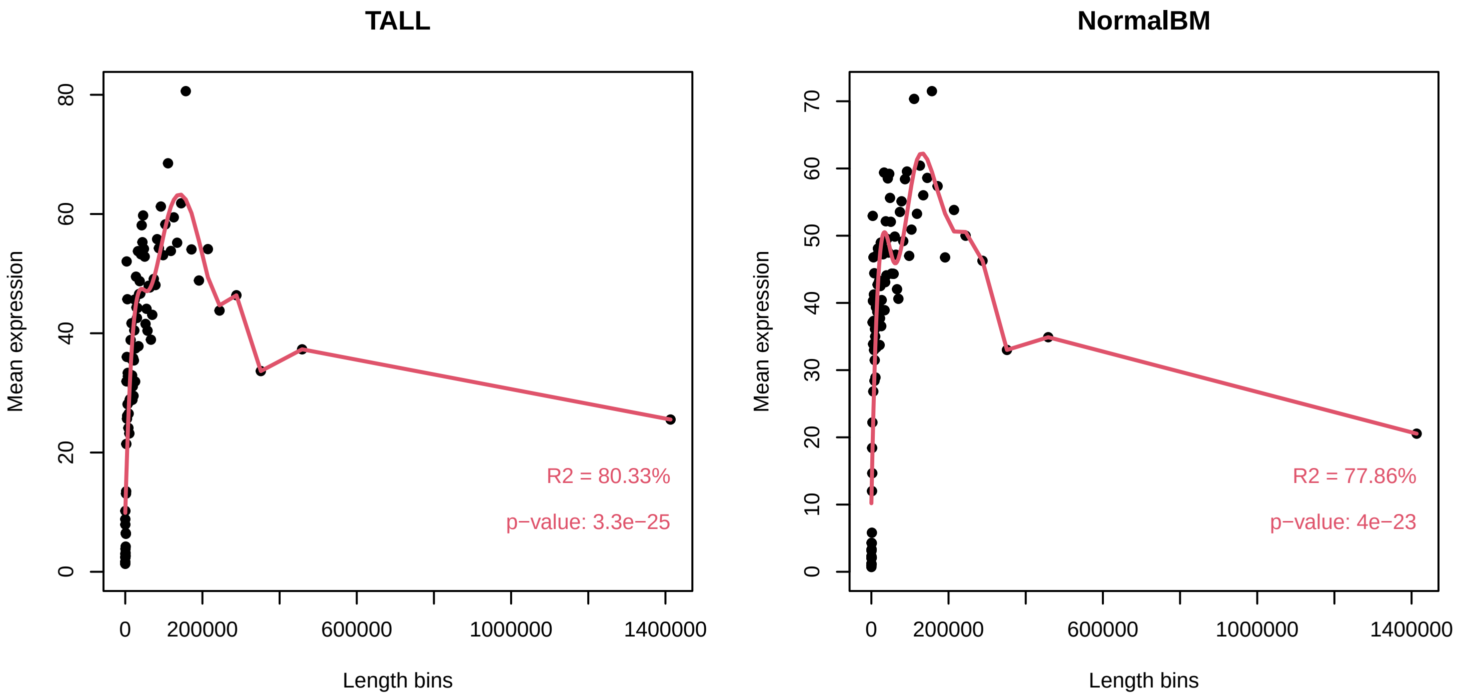

Diagnostic plot for GC content bias

FAILED. At least one of the model p−values was lower than 0.05 and R2 > 70%.

Normalization for correcting GC content bias is recommended.

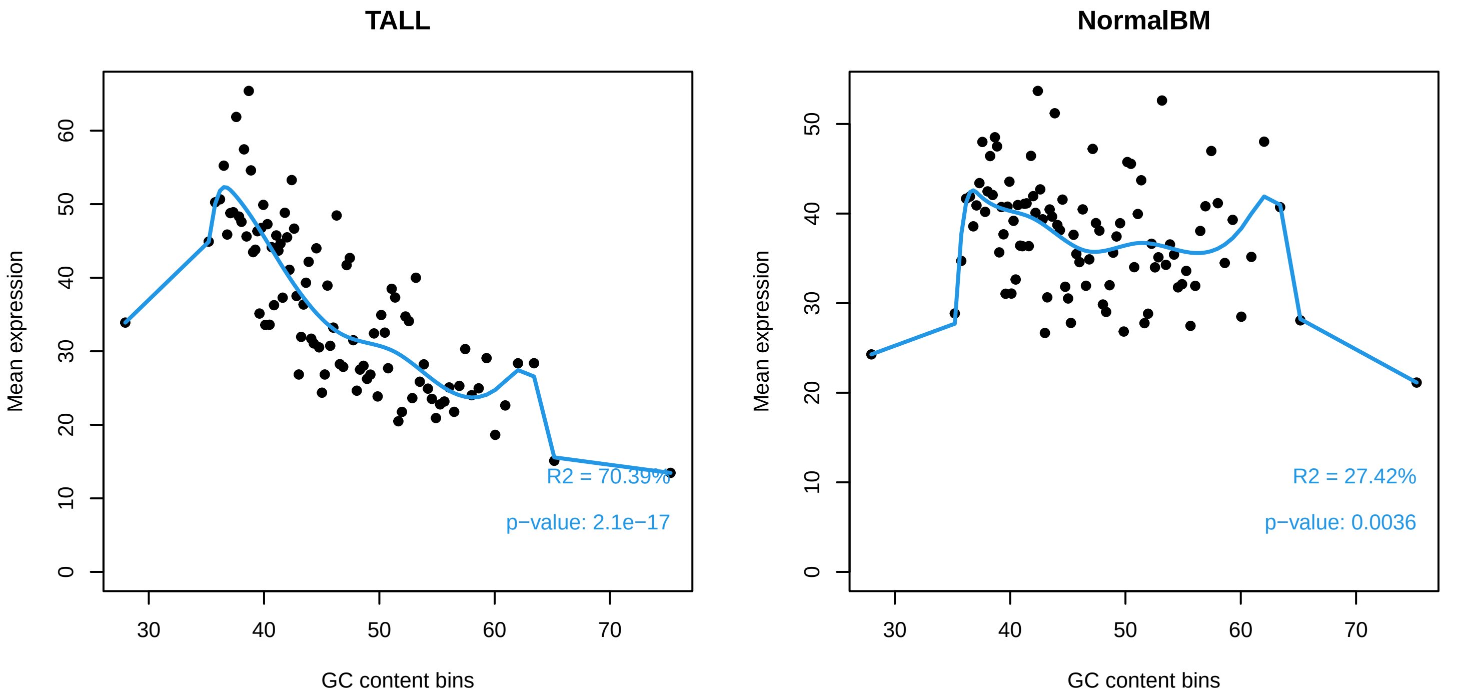

Diagnostic plot for differences in RNA composition

FAILED. There is a pair of samples with significantly different RNA composition

Normalization for correcting this bias is required.

Reference sample: TARGET−10−PATHGY−09A−01R

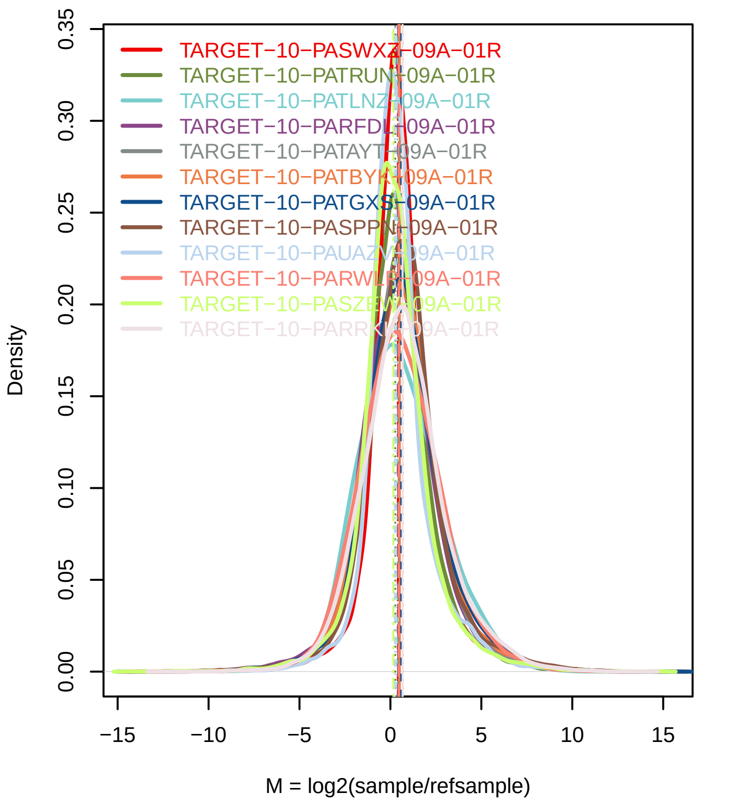

Confidence intervals for median of M values

Sample	0.01%	99.99%	Diagnostic Test
TARGET−10−PASWXZ−09A−01R	[illegible]	0.4491	FAILED
TARGET−10−PATRUN−09A−01R	[illegible]	0.3098	FAILED
TARGET−10−PATLNZ−09A−01R	[illegible]	0.4817	FAILED
TARGET−10−PARFDL−09A−01R	[illegible]	0.4335	FAILED
TARGET−10−PATAYT−09A−01R	[illegible]	0.4944	FAILED
TARGET−10−PATBYK−09A−01R	[illegible]	0.5694	FAILED
TARGET−10−PATGXS−09A−01R	[illegible]	0.6607	FAILED
TARGET−10−PASPPN−09A−01R	[illegible]	0.7211	FAILED
TARGET−10−PAUAZV−09A−01R	[illegible]	0.2842	FAILED
TARGET−10−PARWLP−09A−01R	[illegible]	0.5303	FAILED
TARGET−10−PASZEW−09A−01R	[illegible]	0.1993	FAILED
TARGET−10−PARRKK−09A−01R	[illegible]	0.7265	FAILED
TARGET−10−PAUAYB−09A−01R	[illegible]	−0.0286	FAILED
TARGET−10−PASVPZ−09A−01R	[illegible]	0.3658	FAILED
TARGET−10−PATNIA−09A−01R	[illegible]	−0.0675	FAILED
TARGET−10−PASXMF−09A−01R	[illegible]	0.289	FAILED
TARGET−10−PASKXN−09A−01R	[illegible]	0.1408	PASSED
TARGET−10−PASPBU−09A−01R	[illegible]	0.2934	FAILED
TARGET−10−PARGFL−09A−01R	[illegible]	−0.1581	FAILED
TARGET−10−PATIBE−09A−01R	[illegible]	0.5816	FAILED
TARGET−10−PAUACG−09A−01R	[illegible]	0.771	FAILED
TARGET−10−PATHJF−09A−01R	[illegible]	0.5132	FAILED
TARGET−10−PASLBB−09A−01R	[illegible]	0.6525	FAILED
TARGET−10−PATKYI−09A−01R	[illegible]	0.3981	FAILED
TARGET−10−PASSRP−09A−01R	[illegible]	0.2241	FAILED
TARGET−10−PASUIN−09A−01R	[illegible]	0.3481	FAILED
TARGET−10−PASHXL−09A−01R	[illegible]	0.1345	FAILED
TARGET−10−PARNMV−09A−01R	[illegible]	0.5735	FAILED

Exploratory PCA

Use this plot to see if samples are clustered according to the experimental design.

Use ARSyNseq function to correct potential batch effects.

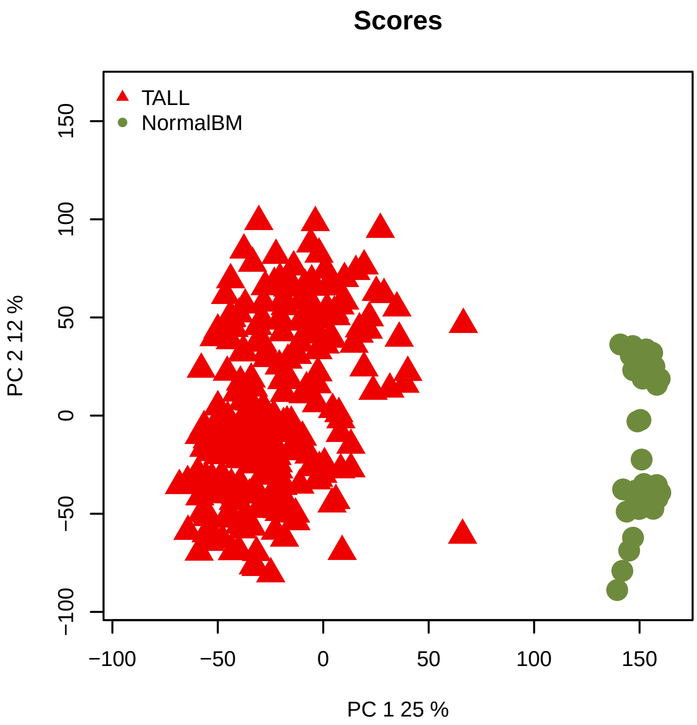

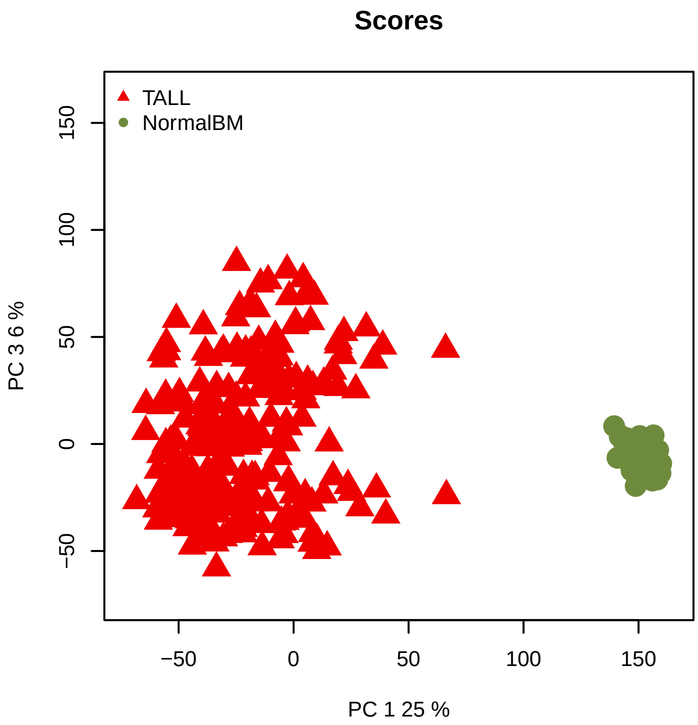

Quality Control of Expression Data
Tall After Normalization

Generated by NOISeq on 21 Nov 2022, 23:59:57

Content

Plot	*Description*
**Biotype detection**	Biotype abundance in the genome with %genes detected (counts > 0) in the sample/condition.<br>Biotype abundance within the sample/condition.
**Biotype expression**	Distribution of gene counts per million per biotype in sample/condition (only genes with counts > 0).
**Saturation**	Number of detected genes (counts > 0) per sample across different sequencing depths
**Expression boxplot**	Distribution of gene counts per million (all biotypes) in each sample/condition
**Expression barplot**	Percentage of genes with >0, >1, >2, >5 or >10 counts per million in each sample/condition.
**Length bias**	Mean gene expression per each length bin. Fitted curve and diagnostic test.
**GC content bias**	Mean gene expression per each GC content bin. Fitted curve and diagnostic test.
**RNA composition bias**	Density plots of log fold changes (M) between pairs of samples.<br>Confidence intervals for the median of M values.
**Exploratory PCA**	Principal Component Analysis score plots for PC1 vs PC2, and PC1 vs PC3.

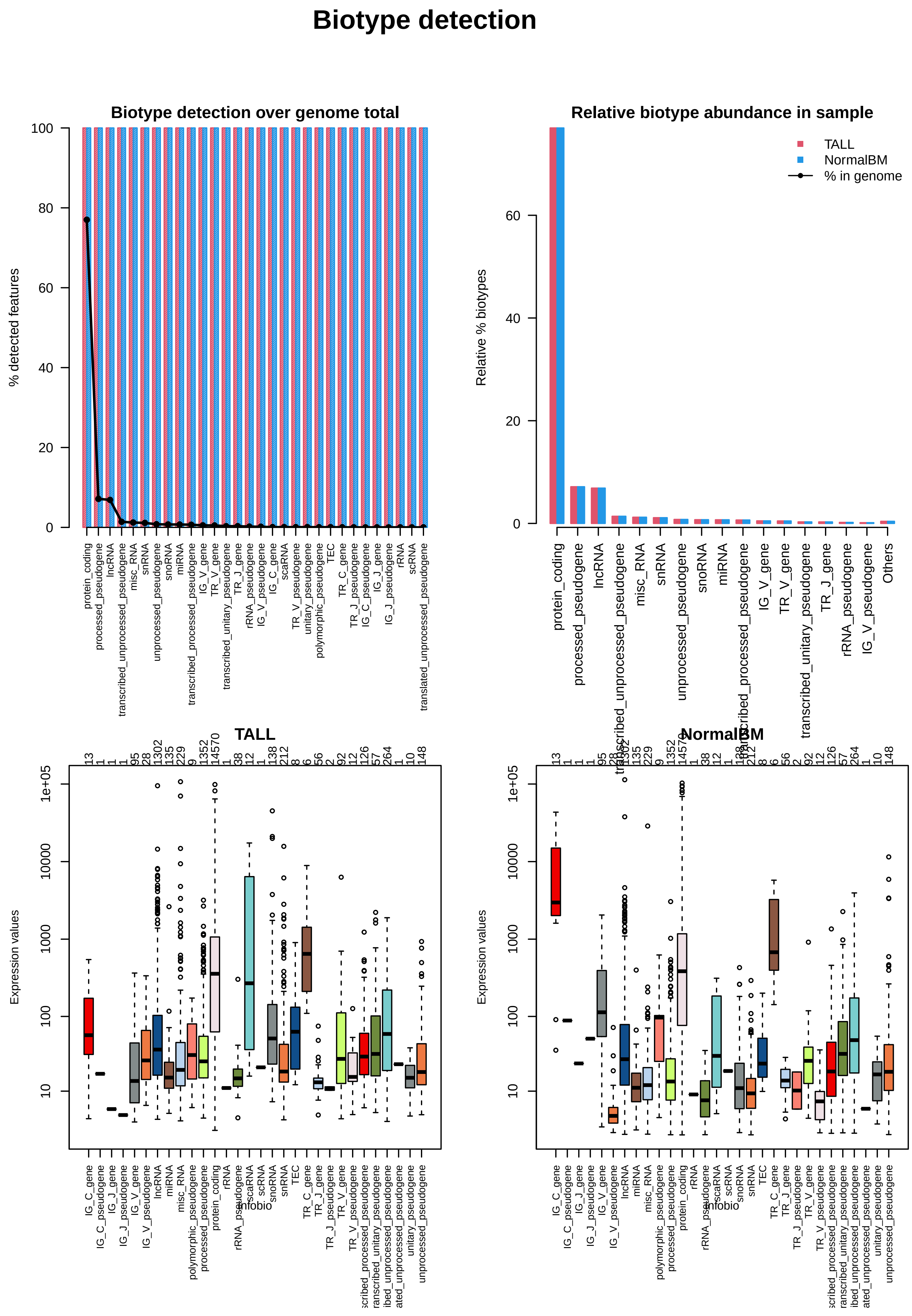
Biotype detection
Biotype detection over genome total
% detected features
protein_coding
processed_pseudogene
lncRNA
transcribed_unprocessed_pseudogene
misc_RNA
snRNA
unprocessed_pseudogene
snoRNA
miRNA
transcribed_processed_pseudogene
IG_V_gene
TR_V_gene
transcribed_unitary_pseudogene
TR_J_gene
rRNA_pseudogene
IG_V_pseudogene
IG_C_gene
scaRNA
TR_V_pseudogene
unitary_pseudogene
polymorphic_pseudogene
TEC
TR_C_gene
TR_J_pseudogene
IG_C_pseudogene
IG_J_gene
IG_J_pseudogene
rRNA
scRNA
translated_unprocessed_pseudogene
Relative biotype abundance in sample
Relative % biotypes
TALL
NormalBM
% in genome
Others
TALL
NormalBM
Expression values
infobio

Sequencing depth & Expression quantification

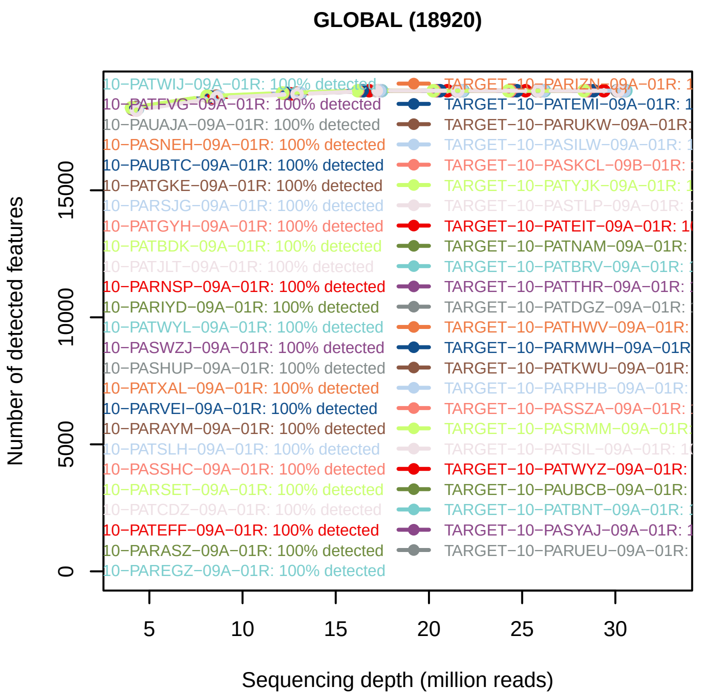

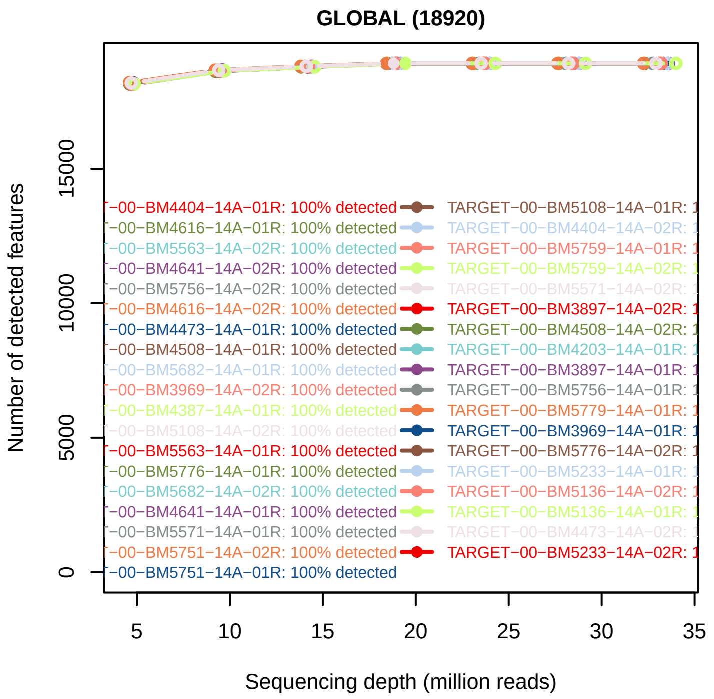

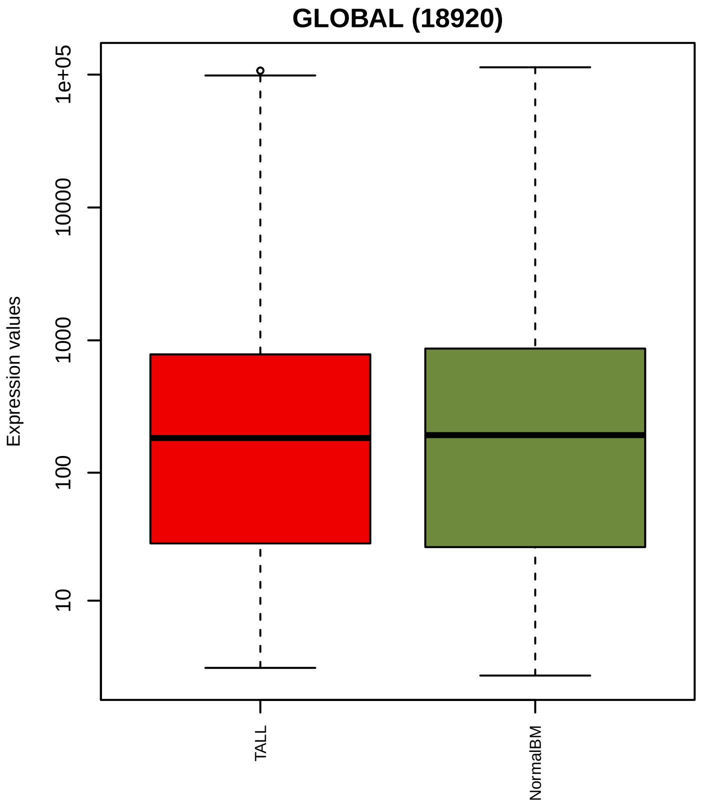

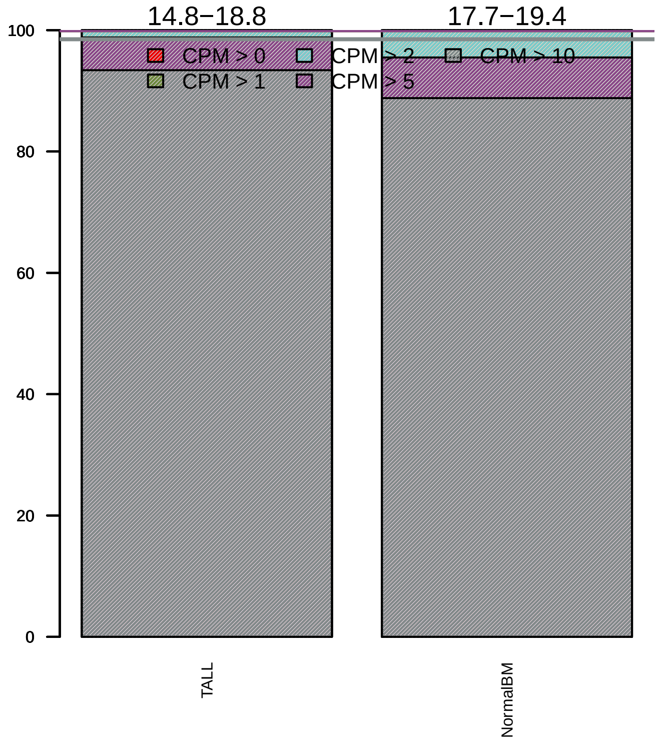

Sequencing bias detection

Diagnostic plot for feature length bias

FAILED. At least one of the model p−values was lower than 0.05 and R2 > 70%.

Normalization for correcting length bias is recommended.

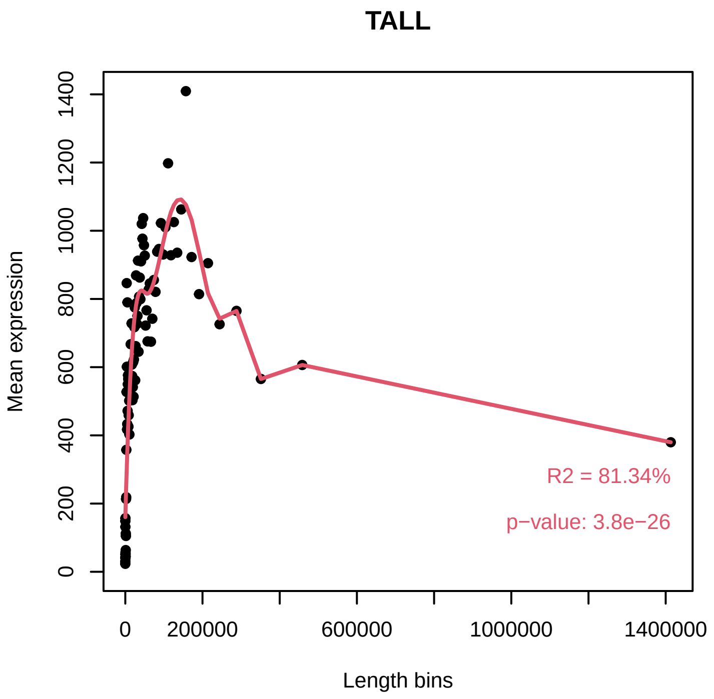

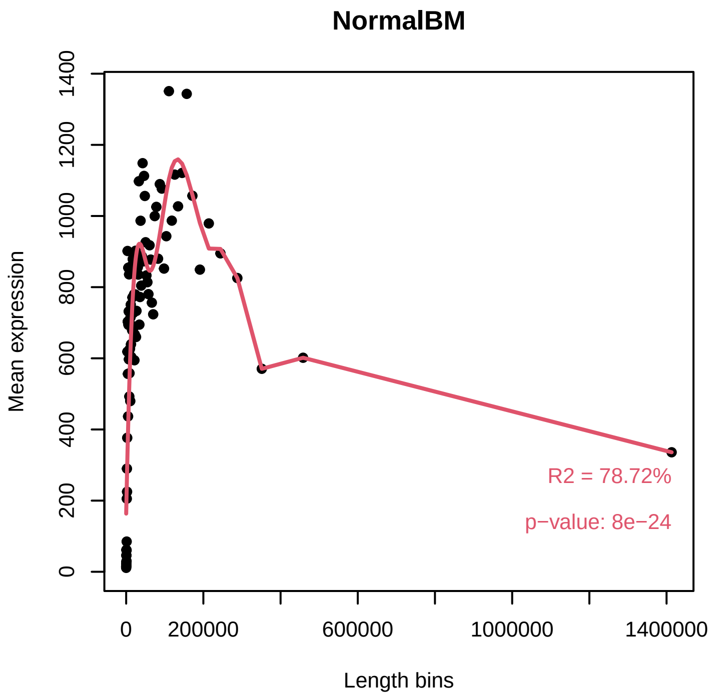

Diagnostic plot for GC content bias

FAILED. At least one of the model p−values was lower than 0.05 and R2 > 70%.

Normalization for correcting GC content bias is recommended.

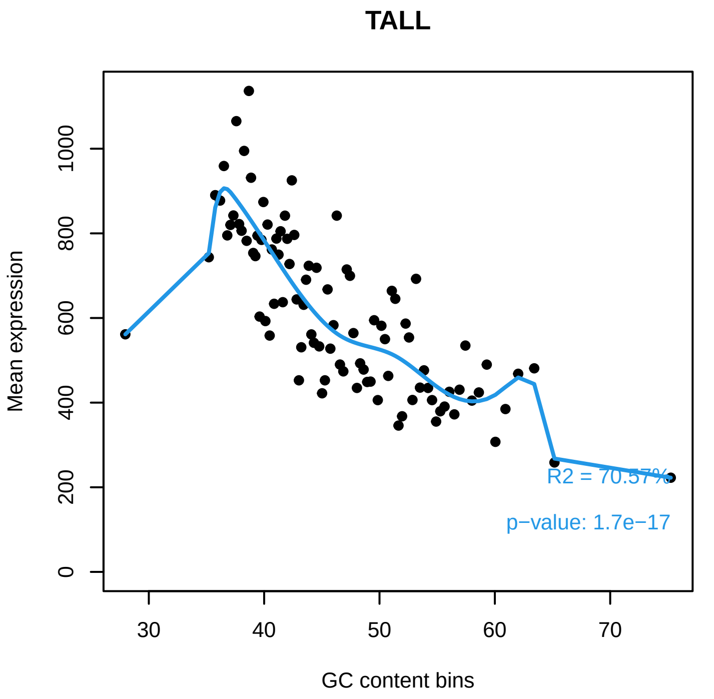

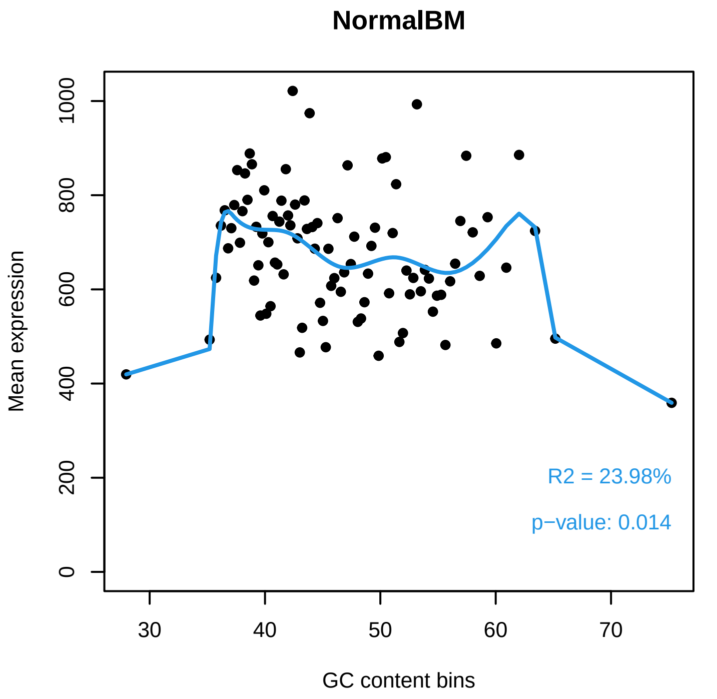

Diagnostic plot for differences in RNA composition

FAILED. There is a pair of samples with significantly different RNA composition

Normalization for correcting this bias is required.

Reference sample: TARGET−10−PATHGY−09A−01R

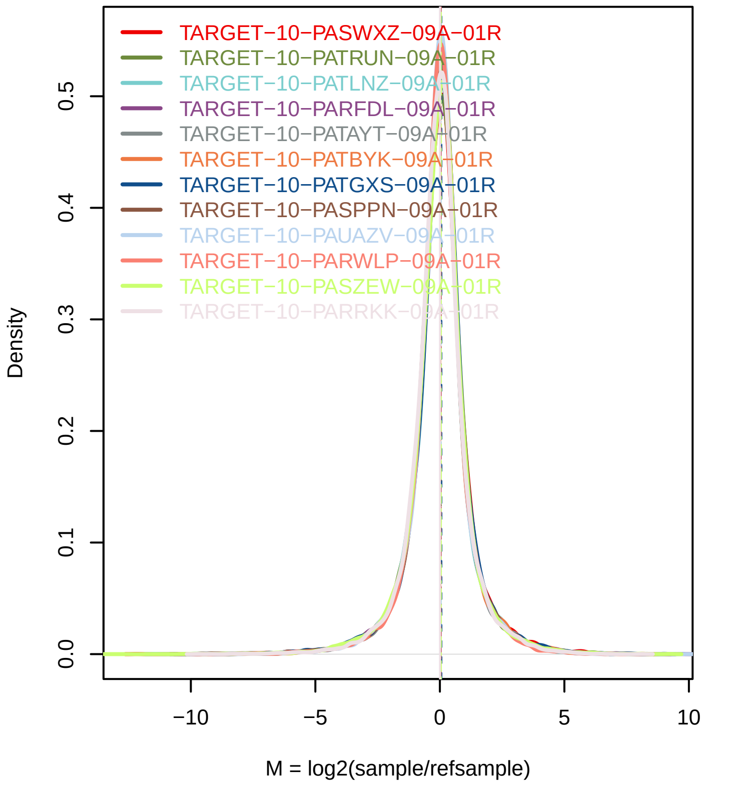

Confidence intervals for median of M values

Sample	0.01%	99.99%	Diagnostic Test
TARGET−10−PASWXZ−09A−01R	0.0291	0.0661	FAILED
TARGET−10−PATRUN−09A−01R	0.0223	0.0644	FAILED
TARGET−10−PATLNZ−09A−01R	0.0089	0.0568	FAILED
TARGET−10−PARFDL−09A−01R	0.0159	0.0554	FAILED
TARGET−10−PATAYT−09A−01R	0.011	0.0459	FAILED
TARGET−10−PATBYK−09A−01R	−0.0014	0.0374	PASSED
TARGET−10−PATGXS−09A−01R	0.0368	0.0759	FAILED
TARGET−10−PASPPN−09A−01R	0.0048	0.0469	FAILED
TARGET−10−PAUAZV−09A−01R	0	0.0389	FAILED
TARGET−10−PARWLP−09A−01R	0.0053	0.0424	FAILED
TARGET−10−PASZEW−09A−01R	0.0192	0.0577	FAILED
TARGET−10−PARRKK−09A−01R	−0.0143	0.0267	PASSED
TARGET−10−PAUAYB−09A−01R	−0.0086	0.0337	PASSED
TARGET−10−PASVPZ−09A−01R	0.0479	0.0921	FAILED
TARGET−10−PATNIA−09A−01R	−0.0158	0.0251	PASSED
TARGET−10−PASXMF−09A−01R	0.0082	0.046	FAILED
TARGET−10−PASKXN−09A−01R	−0.0099	0.0356	PASSED
TARGET−10−PASPBU−09A−01R	0.0038	0.0473	FAILED
TARGET−10−PARGFL−09A−01R	−0.0183	0.0185	PASSED
TARGET−10−PATIBE−09A−01R	0.0284	0.0703	FAILED
TARGET−10−PAUACG−09A−01R	0.015	0.0582	FAILED
TARGET−10−PATHJF−09A−01R	−0.0106	0.0272	PASSED
TARGET−10−PASLBB−09A−01R	−0.0086	0.0359	PASSED
TARGET−10−PATKYI−09A−01R	0.0077	0.0504	FAILED
TARGET−10−PASSRP−09A−01R	0.0116	0.0518	FAILED
TARGET−10−PASUIN−09A−01R	0.0125	0.0538	FAILED
TARGET−10−PASHXL−09A−01R	−0.0038	0.0401	PASSED
TARGET−10−PARNMV−09A−01R	0.0205	0.0662	FAILED

Exploratory PCA

Use this plot to see if samples are clustered according to the experimental design.

Use ARSyNseq function to correct potential batch effects.

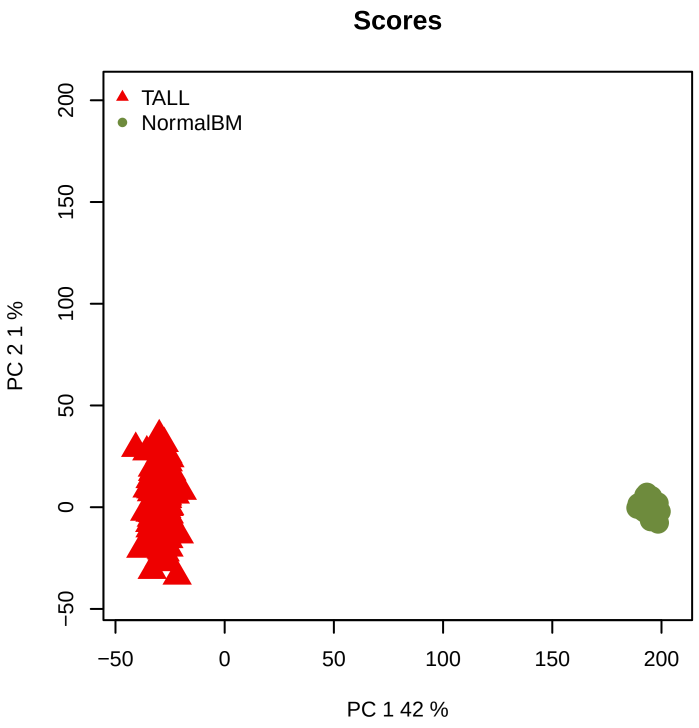

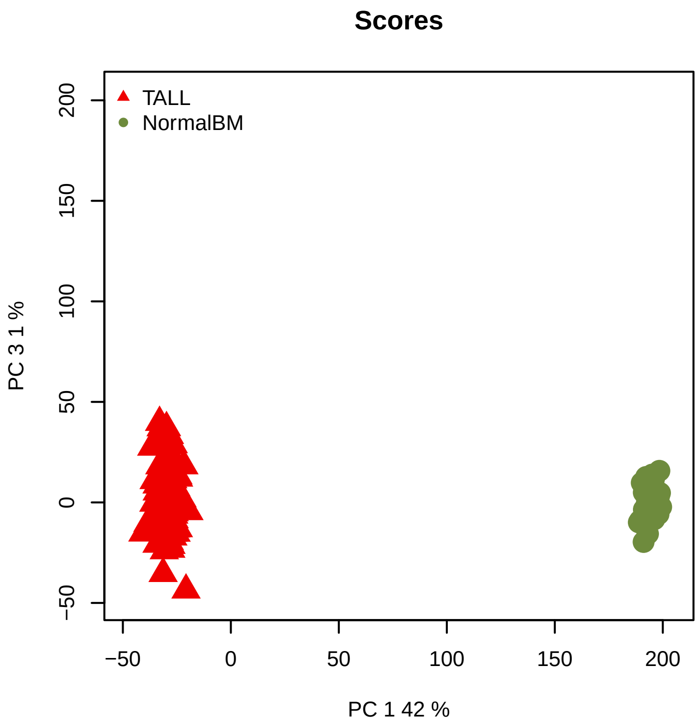

Supplement: Supplementary file 5 — Supplementary Information 5. [file 41598_2023_46655_MOESM5_ESM.pdf]
