# Supplementary material for: The network structure of hematopoietic cancers
Source: Sci Rep. 2023 Nov 13;13:19837. doi: 10.1038/s41598-023-46655-2 (PMC10645882; doi:10.1038/s41598-023-46655-2)
Supplement: Supplementary file 6 — Supplementary Legends. [file 41598_2023_46655_MOESM6_ESM.docx]

Supplementary material

1. Network files for the top-100,000 interactions in the five phenotypes.

2. Kolmogorov-Smirnov p-values for the comparison between fraction of intra-chromosomal interactions in normal and cancer tissues at different MI thresholds.

3. Differential gene expression analysis for all hematopoietic cancers.

4. Top: Bipartite networks of communities and enriched biological processes. Bottom: Shared interactions between MI and Spearman networks.

5. Quality control for data pre-processing.
